# 3D-QSAR and free energy landscape analysis for predicting novel JAK3 inhibitors in rheumatoid arthritis therapy

**DOI:** 10.1038/s41598-026-50981-6

**Published:** 2026-04-29

**Authors:** Fatemeh Rahimi, Maryam Abbasi

**Affiliations:** 1 Student Research Committee, Faculty of Pharmacy, Hormozgan University of Medical Sciences, Bandar Abbas, Iran; 2 Endocrinology and Metabolism Research Center, Hormozgan University of Medical Sciences, Bandar Abbas, Iran; 3 Department of Pharmaceutical Chemistry, School of Pharmacy, Hormozgan University of Medical Sciences, Bandar Abbas, Iran

**Keywords:** Rheumatoid arthritis, JAK3 inhibitor, Molecular dynamics simulation, Free energy landscape, MM-GBSA, Docking, Computational biology and bioinformatics, Drug discovery, Structural biology

## Abstract

JAK3 is a main tyrosine kinase within the JAK kinase family, whose inhibition plays a crucial role in the treatment of rheumatoid arthritis (RA). Multiple selective JAK3 inhibitors with different scaffolds exist. Decernotinib, a pyrrolopyridine-based RA candidate, showed early potential but was discontinued during Phase II. Currently, no selective JAK3 inhibitor is available for rheumatoid arthritis. In this research, an integrated ligand- and structure-based approach is employed to develop novel JAK3 inhibitors. Two 3D-QSAR models were developed based on CoMFA and CoMSIA methods, using a library of pyrimidine-based JAK3 inhibitor structures. The best models have been chosen based on statistical values of r² greater than 0.85 for CoMFA and CoMSIA. The models underwent internal and external validation. The electrostatic, steric, hydrogen bond acceptor, hydrogen bond donor, and hydrophobic contour maps using CoMFA and CoMSIA methods were generated, and 103 novel compounds were predicted based on all of these features. To explore the binding orientations and the associated binding free energies of the predicted compounds within JAK3’s active site, molecular docking followed by MM-GBSA analyses was conducted. Molecular dynamics simulations were additionally performed to assess the results for the top five predicted compounds. Among the predicted compounds, compound 56 stood out as the most promising candidate, displaying superior binding stability. Additionally, the free energy landscape for the JAK3-56 complex revealed stable conformations. Generally, 3D-QSAR pharmacophore modeling and MD observations will inform lead optimization and the creation of new, effective, selective JAK3 inhibitors.

**Supplementary Information:**

The online version contains supplementary material available at 10.1038/s41598-026-50981-6.

## Introduction

Rheumatoid arthritis (RA) is a persistent autoimmune disorder marked by joint pain and inflammation, most notably affecting the hands and feet. It may also affect the heart, lungs, and skin^1,2^. The worldwide prevalence of RA varies between 0.4% and 1.3%, with a higher incidence in women compared to men^3^. Treatment options for RA include NSAIDs, glucocorticoids, and DMARDs. DMARDs, or disease-modifying anti-rheumatic drugs, are divided into biologic, conventional synthetic, and targeted synthetic categories. Targeted synthetic DMARDs specifically influence certain molecules or pathways in the body, such as inhibitors of phosphodiesterase-4, tyrosine kinase, and Janus kinases, representing a notable subgroup^4^.

**Fig. 1:**
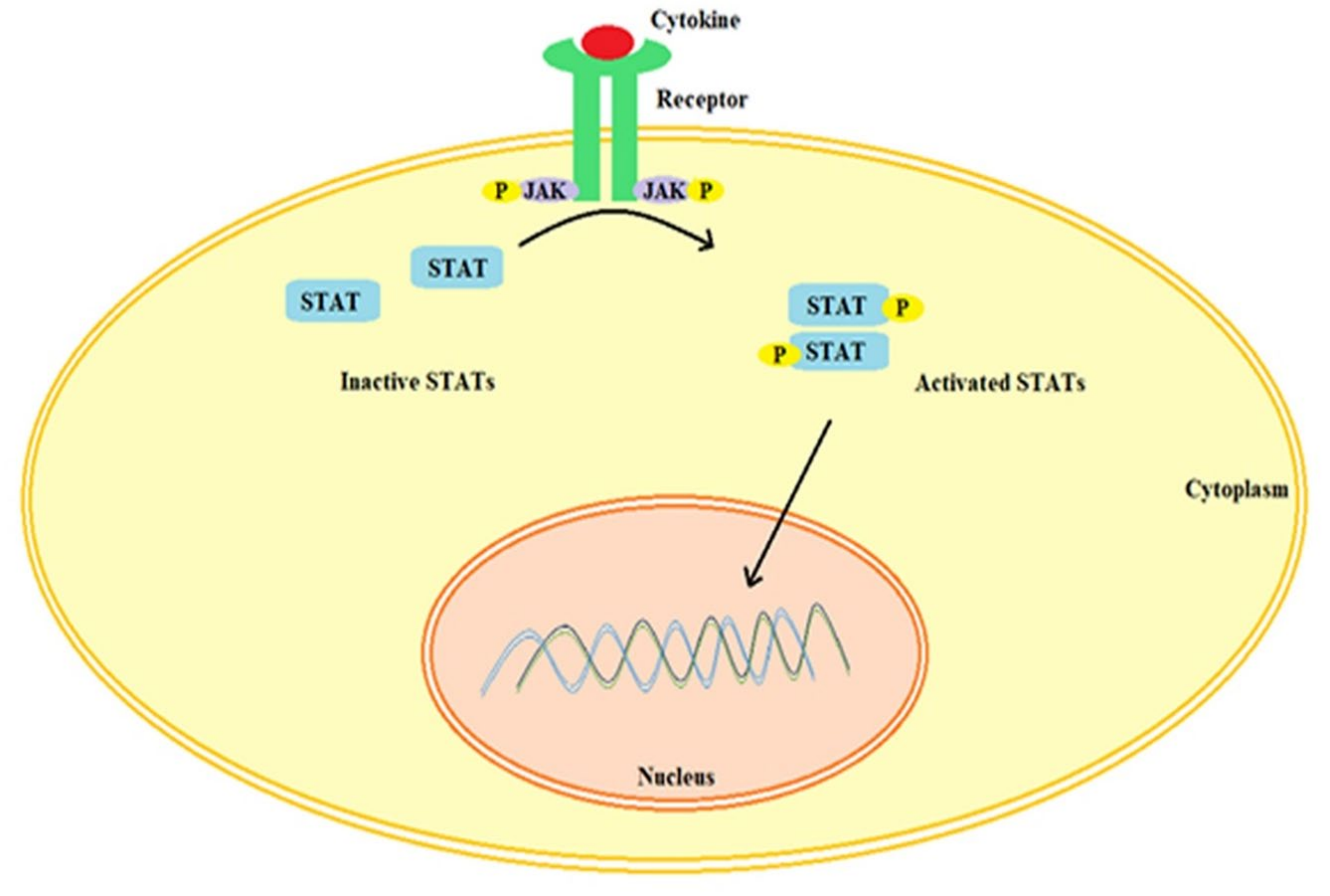
JAK-STAT signaling pathway. A cytokine that binds to its receptor triggers the activation of JAK proteins. Following this, JAKs participate in a phosphorylation process, wherein they phosphorylate each other, as well as the receptor chains. This phosphorylation event establishes a binding site for signal transducer and activator of transcription (STAT) molecules. After being phosphorylated by the JAKs, the STATs detach from the receptor. The phosphorylated STATs subsequently dimerize, migrate to the cytoplasm, and then enter the nucleus to regulate specific target genes.

The Janus kinase (JAK) family constitutes a group of non-receptor tyrosine kinases present in the human body, playing a crucial role in the growth, survival, evolution, and differentiation of cells. Janus kinases are vital for triggering immune and inflammatory responses through cytokine-dependent signaling pathways and transcriptional regulation. The JAK-STAT signaling pathway is illustrated in Fig. 1^5,6^.

The JAK family in mammals consists of four members: JAK1, JAK2, JAK3, and tyrosine kinase 2. JAK3 is predominantly expressed in hematopoietic cells and is activated by gamma-chain cytokines, including interleukin 2, 4, 7, 9, 15, and 21^5,6^. A defect in JAK3 results in diminished immune T cell proliferation, decreased dendritic cell differentiation, and reduced synthesis of gamma interferon, which ultimately leads to severe combined immunodeficiency. Conversely, heightened JAK3 activity is associated with various cancers, malignancies, and autoimmune disorders such as rheumatoid arthritis^7,8^.

**Fig. 2:**
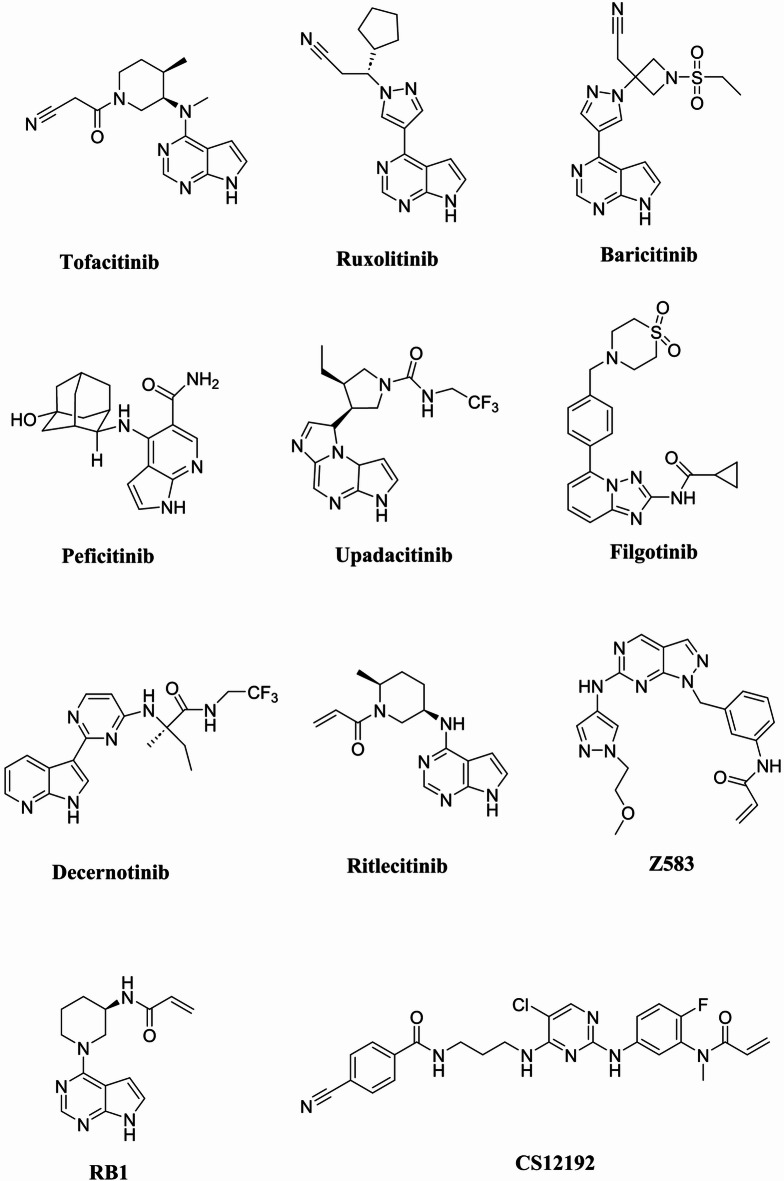
Chemical structure of JAK inhibitors.

Recent advancements in JAK inhibitors are focused on treating inflammatory and immune disorders, with numerous candidates undergoing trials. First-generation non-selective JAK inhibitors, including Tofacitinib, Ruxolitinib, Baricitinib, and Peficitinib, have shown safety and efficacy; however, their extensive inhibitory effects on cytokines have resulted in adverse effects^9^. Consequently, next-generation selective JAK inhibitors have emerged, such as Upadacitinib and Filgotinib (targeting JAK1), as well as Decernotinib and Ritlecitinib (targeting JAK3)^5,10^. Within the JAK family, JAK3 is pivotal in immune diseases like rheumatoid arthritis (RA), which is associated with gamma chain cytokines. Ritlecitinib, featuring a pyrrolopyrimidine scaffold, is the latest FDA-approved irreversible JAK3 selective inhibitor and is currently undergoing phase 2b/3 trials for alopecia areata^11^. Decernotinib, based on a pyrrolopyridine structure and designed for RA, demonstrated potential in initial studies but was discontinued during phase II trials^12^. Furthermore, there are additional JAK3 selective inhibitors in preclinical stages or under investigation, primarily based on pyrimidine (CS12192), pyrazolopyrimidine (Z583), and 4-aminopiperidine (RB1) (refer to Fig. 2). It is important to note that there are currently no JAK3 selective inhibitors available on the market for RA^9,13,14^.

JAK3 contains a unique Cys909 residue that substitutes for serine found in other JAK isoforms, rendering Cys909 an attractive target for selective and irreversible inhibition. Ritlectinib covalently binds Cys909, functioning as a selective, irreversible JAK3 inhibitor. The JAK3 binding pocket comprises a mix of hydrophobic and hydrophilic amino acids, in addition to structural water molecules. Key residues contributing to hydrogen bonding include Leu905 and Asp967 as donors, and Glu903 and Arg953 as acceptors. Van der Waals interactions involve Val836, Gly829, Leu956, Asn954, and Leu828, with polar contacts observed for Asp967 and Arg953 within the active site. Hydrophobic contacts are formed by Val844, Lys855, Val836, Met902, and Gly969^10,15^. Theoretical drug design techniques offer significant advantages in terms of cost and time compared to experimental methods, as they enable researchers to forecast drug interactions before synthesis occurs^16^. Prominent strategies encompass ligand-based approaches, including pharmacophore modeling and quantitative structure-activity relationship (QSAR), which correlate chemical structures with biological activity, thereby identifying potential drug candidates from pre-existing compounds^17^. On the other hand, structure-based techniques, such as molecular docking and dynamics simulations, utilize the three-dimensional structures of target proteins to develop and enhance new binding molecules^18^. Additionally, virtual screening expedites the drug discovery process by swiftly evaluating extensive compound libraries, facilitating the identification of promising candidates^19^. Currently, some studies contribute to JAK3 inhibitor discovery by applying CADD techniques, including 3D-QSAR analyses, molecular dynamics, and Structure-Based Virtual Screening (SBVS) strategies^20–22^. In this instance, virtual screening of existing FDA-approved drugs revealed three JAK3 inhibitors, such as lomitapide, ledipasvir, and venetoclax, with prospective applicability to rheumatoid arthritis management^23^.

This investigation is focused on the development of bioinformatic models for pyrimidine-based compounds, extending previous research related to Janus kinase 3 (JAK3) inhibitors (references^6,24,25^). This study focuses on developing 3D-QSAR models to predict novel JAK3-selective inhibitors, employing molecular docking coupled with molecular dynamics (MD) simulations. The 3D-QSAR model was employed to generate contour maps for predicting activities and to assess enhancements and reductions in activity for the modified lead molecules. The predicted compounds, derived from 3D-QSAR models, were prioritized based on docking scores, binding free energies, and molecular interactions. The best-performing lead molecules were evaluated for their stability against JAK3 by subjecting them to MD simulations.

## Methodology

### Dataset creation for 3D-QSAR modeling

QSAR analysis is a widely utilized computational approach in the rational design of novel JAK3 inhibitors. QSAR models aim to predict the biological activity of new compounds based on existing structure-activity data. However, traditional QSAR methods are limited in their capacity to account for the three-dimensional (3D) conformational features of molecules, which are crucial for accurately modeling molecular interactions^26^. To overcome this limitation, three-dimensional QSAR (3D-QSAR) techniques have been developed, integrating spatial molecular properties to enhance predictive accuracy. Notable 3D-QSAR methodologies include Comparative Molecular Field Analysis (CoMFA) and Comparative Molecular Similarity Index Analysis (CoMSIA). Both approaches utilize 3D descriptors, primarily steric and electrostatic fields, to establish quantitative relationships between molecular structure and biological activity, thereby improving the reliability of activity predictions^27^. The CoMSIA method further advances the modeling process by incorporating a broader set of molecular fields, including hydrogen bond acceptor, hydrogen bond donor, steric, electrostatic, and hydrophobic fields, which collectively contribute to a more comprehensive and robust predictive model^28^.

A total of 95 pyrimidine-based JAK3 inhibitor structures, with IC_50_ values ranging from 5 to 8.77 nM, were selected from three distinct sources (Table S1)^6,24,25^. The IC_50_ data were converted to their negative logarithmic form, pIC_50_, to facilitate quantitative correlation. The dataset was randomly split into an 80-compound training set for developing the 3D-QSAR model and a 15-compound test set for validation of the model’s robustness and its ability to predict biological activity.

All 95 compounds were subsequently subjected to spatial structure optimization using SYBYL-X 2.0 software^29^. The optimization utilized the Tripos force field in conjunction with Gasteiger-Hückel charges, applying a non-bonded cutoff distance of 8.00 Å and a dielectric constant of 1.00. The Powell gradient algorithm was utilized to perform the energy minimization.

### Compounds alignment and 3D-QSAR model creation

In 3D-QSAR, the compounds’ structural alignment is a main step that directly influences the accuracy of further biological activity predictions. Proper overlay of the compounds ensures their spatial comparability within the modeling process. In this study, the alignment was carried out based on the pyrimidine ring, with the most active compound (12(A)) serving as a reference template. This approach facilitates consistent and precise alignment of all compounds relative to this key molecule. By using the most active compound as a reference benchmark for overlay, the identification of structural features that contribute to biological activity is significantly improved. This information is invaluable for guiding future drug design and optimization efforts. CoMFA and CoMSIA descriptors were chosen separately.

3D-QSAR models were created using the CoMFA/CoMSIA methodologies. Contour maps for steric and electrostatic properties were produced for both the CoMFA/CoMSIA methodologies. Additionally, the hydrophobic field, as well as the hydrogen bond donor and acceptor fields, were derived using the CoMSIA methodology. A PLS-based quantitative correlation model was established to link grid-point molecular field values with affinity measurements. The optimal number of principal components was determined from the results of Leave-One-Out cross-validation, and a 3D-QSAR model was subsequently developed using these components, which demonstrated the best performance. The model was constructed and then utilized to predict the affinities of the test-set compounds.

### Application domain (AD) and Y randomization test

Evaluating the applicability domain (AD) for QSAR models is a crucial step to ensure reliability, especially for structurally similar compounds, where the model’s predictive scope must be carefully assessed. In this context, the AD is defined using the leverage approach combined with standardized procedures to guarantee prediction accuracy. In QSAR modeling, leverage is a frequently employed statistical measure to gauge the impact of single observations on the model^30^. Elevated leverage suggests samples with unique or outlier features that substantially influence the structure–activity relationship and the resulting model performance. Assessing whether leverage exceeds a specified cutoff allows appraisal of the model’s stability in the domain and the trustworthiness of predictions. The calculation of the threshold is described by the equation.

$$\:{h}^{*}={3p}^{{\prime\:}}/n$$

In the presented equation, $\:{p}^{{\prime\:}}$ is the descriptor value increased by one, and n is the count of the training set.

Additionally, to improve model robustness and guard against incidental correlations, a Y-randomization analysis was carried out to substantiate the non-randomness of the results^31^.

### Molecular docking

The binding interactions between a ligand and a biological macromolecule receptor are modeled computationally through molecular docking^18^.

The anticipated interactions and binding energies of the predicted compounds with JAK3 were analyzed through molecular docking, employing Schrodinger 2015 software^32^. The chosen structure among human JAK3 experimental X-ray conformations was the PDB entry 4Z16, resolved at 2.90 Å^15^. The preparation of the protein was carried out in two distinct phases using the protein preparation module. Initially, the removal of the co-crystallized ligand, water molecules, and chains B, C, and D was performed, followed by the optimization of the protein at a pH of 7.4 using the OPLS-2005 force field. A grid box possessing a grid-point spacing of 25 Å was generated at the coordinate location (-6.662584, -14.305975, -1.231545) for the docking study. In addition to the active site, the grid box encompassed significant areas of the surrounding surface. The three-dimensional conformations of all designed compounds were visualized in Schrodinger 2015. Ligand preparation was performed in LigPrep module^32^ utilizing the OPLS-2005 force field at a pH of 7.4 ± 0.5. Both the grid receptor and the optimized ligand were subsequently imported into the ligand-docking window, after which the extra-precision (XP) docking procedure was performed.

### Binding free energy estimation

The binding free energy was calculated by inputting the docking outcomes into the MM-GBSA method, taking into account the presence of the solvate, and utilizing the OPLS-2005 force field in conjunction with the VSGB solvation model as implemented in Schrodinger 2015^32^.

### MD simulation

The protein–predicted ligand complexes with more negative binding free energy underwent MD simulations in GROMACS 2021.5 to explore conformational rearrangements and atomistic motion^33^. The extracted ligands from the best docking outcomes were optimized using UCSF CHIMERA with Amber99.SB force field and subsequently were introduced to the ACPYPE package to create a ligand topology file^34,35^. The protein topology file was generated utilizing the Amber99.SB force field along with the TIP3P water model. The ligand-protein complex was placed within a dodecahedron-shaped box, maintaining a minimum separation of 1 nm between the protein surface and the boundaries of the box, which housed around 10,389 solvent molecules. To neutralize the overall charge of the system, the solvent water molecules were replaced with CL ions.

First, steepest-descent minimization was used to minimize the system energy, followed by refinement with conjugate-gradient minimization. Long-range electrostatic interactions were treated by PME, and Lennard-Jones and Coulomb interactions were constrained via LINCS with a 1 Å cutoff.

After the energy minimization process, the system underwent equilibration under both NVT and NPT conditions to guarantee the stabilization of volume, pressure, and temperature. This process commenced with a 500 ps phase at constant volume and temperature (NVT), during which a temperature of 300 K was attained using a V-rescale thermostat. Following this, the system transitioned to a constant volume and constant pressure (NPT) phase lasting 500 ps, achieving a pressure of 1 atm with the aid of a Berendsen barostat. Subsequently, a molecular dynamics production run of 100 ns was conducted, with coordinates being recorded at regular intervals of 2 fs. After the simulations were completed, trajectory analysis was performed using tools available in the Gromacs suite. A range of analyses was calculated, including radius of gyration (Rg), root mean square fluctuation (RMSF), root mean square deviation (RMSD), and the number of hydrogen bonds.

### Free binding energy landscape analysis

The PCA-driven free binding energy landscape (FEL) analysis permits the depiction of stable protein conformations within a ligand-bound complex, using Gibbs free energy through conformational mapping and thermodynamics-based energy calculations^36^. This study employed the first two principal components (PC1 and PC2) derived from each simulation for the FEL computation. In conjunction with PC1 and PC2, the GROMACS g_sham script was utilized, using Boltzmann inversion to construct a multidimensional histogram for the computation of Gibbs free energy for each complex.

### The dynamic cross-correlation matrix analysis

The dynamic cross-correlation matrix (DCCM) constitutes a quantitative representation of the temporal co-movement between pairs of atoms, specifically the Cα atoms of amino acid residues, throughout a molecular dynamics (MD) trajectory. The element at position (i, j) in the DCCM quantifies the degree to which the movements of the Cα coordinates of residues i and j are correlated over the trajectory. The correlation values typically span from + 1 to − 1, where + 1 denotes perfect positive correlation, meaning the two residues move in the same direction simultaneously. 0 denotes no correlation, indicating independent motions. −1 denotes perfect negative (anticorrelated) correlation, meaning the residues move in opposite directions. Here, the DCCM analysis was conducted for each simulation.

### Probability density function analysis

The probability density function (PDF) analysis employs a kernel density estimate (KDE) approach to quantify the likelihood, or frequency, of events within an MD simulation trajectory^37^.

## Results and discussion

### Statistical results obtained from the CoMFA and CoMSIA models

**Fig. 3:**
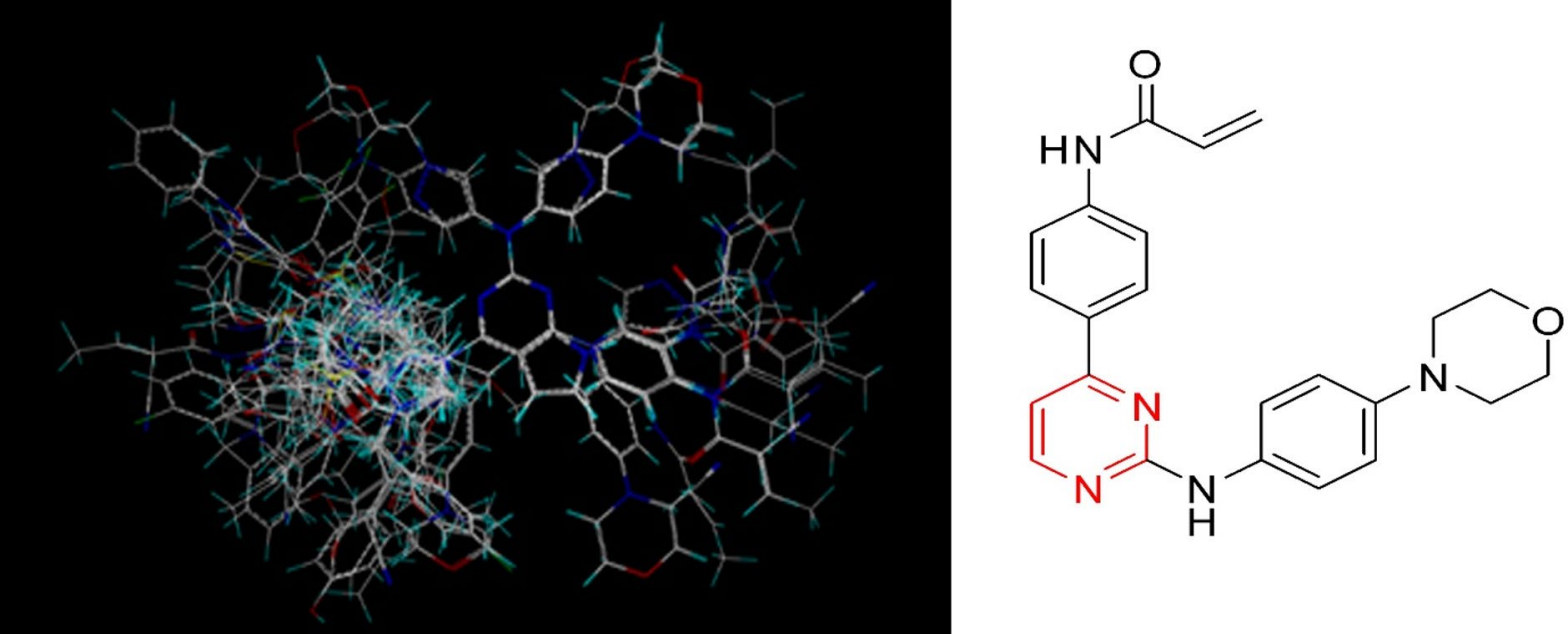
The alignment of all studied compounds on the pyrimidine ring of compound 12(A), as the template molecule.

In this study, compound 12(A) served as the template, with geometrically optimized molecules aligned on the pyrimidine ring through database alignment in SYBYL-X 2.0 (Fig. 3).

All compounds were divided into two groups: training and testing. The CoMFA and CoMSIA models were developed using the training group and subsequently validated with the testing group. The relationship between CoMFA/CoMSIA descriptors and biological activity was assessed using partial least squares (PLS) regression. The optimal CoMFA and CoMSIA models were used to derive the activities and residuals for all compounds (Table S2). Figure S1 presents the correlation between predicted and empirical activities as derived from the CoMFA and CoMSIA models, highlighting the agreement between the two modeling approaches. To validate the model internally, r² and the standard error of estimate were computed, and cross-validated q² was used to assess model quality. A 2.00 kcal/mol column filter was applied to streamline analysis and mitigate noise^38^. The mean absolute error was calculated by comparing the predicted efficacy of molecules with their actual efficacy using the following equation^39^:

$$\:\:\mathrm{M}\mathrm{A}\mathrm{E}=\frac{{\sum\:}_{\mathrm{i}=1}^{\mathrm{N}}\left|\right({\mathrm{p}\mathrm{I}\mathrm{C}}_{50\:\mathrm{a}\mathrm{c}\mathrm{t}\mathrm{u}\mathrm{a}\mathrm{l}\:}-{\mathrm{p}\mathrm{I}\mathrm{C}}_{50\:\mathrm{p}\mathrm{r}\mathrm{e}\mathrm{d}\mathrm{i}\mathrm{c}\mathrm{t}\mathrm{e}\mathrm{d}}\left)\right|}{\mathrm{N}}$$

**Fig. 4:**
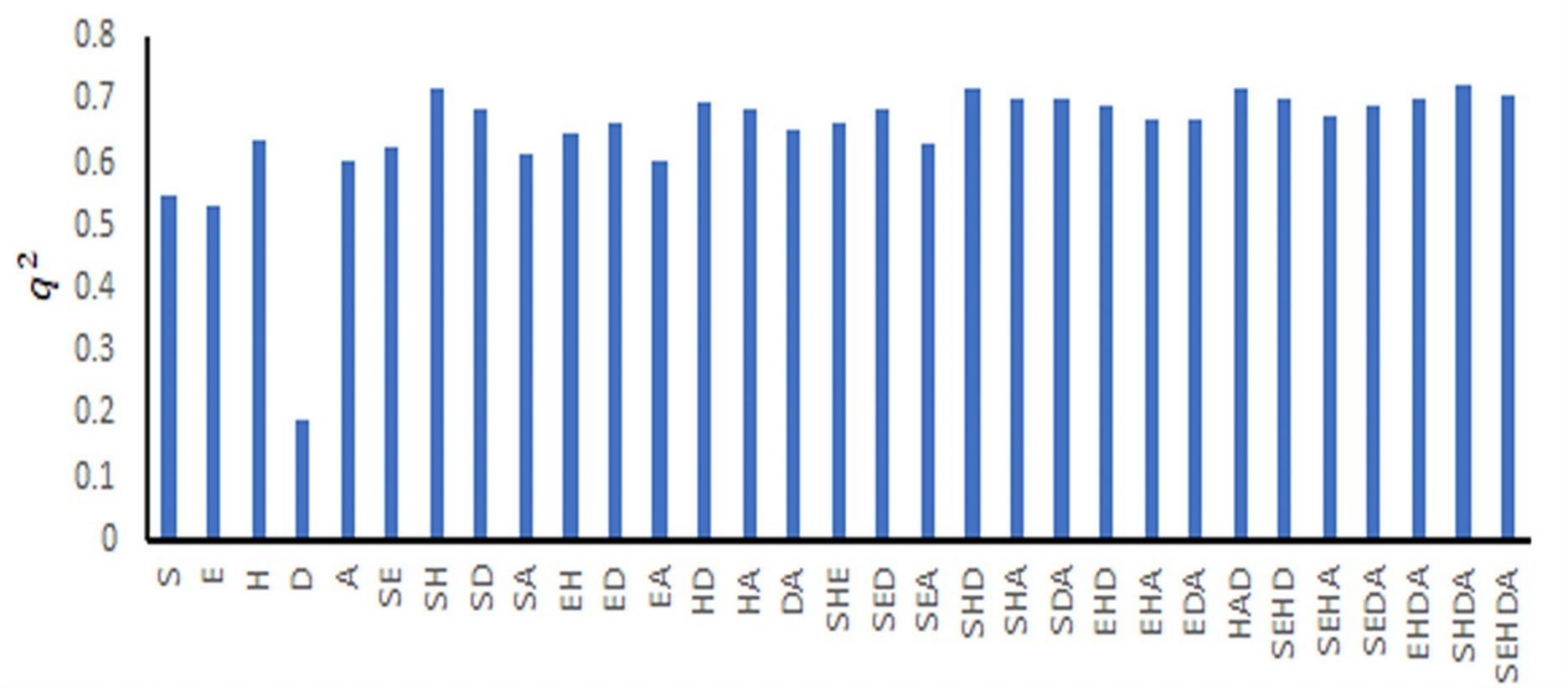
The q² values pertain to the 31 possible descriptor combinations in the CoMSIA model. (S for steric, H for hydrophobic, E for electrostatic, and A/D for H-bond acceptor /donor).

In the CoMFA analysis, two distinct fields, steric and electrostatic, contribute at different levels (21.71% and 78.27%, respectively). For the steric/electrostatic CoMFA model, the q², r², MAE, and SEE values were determined, with the optimal number of components (ONC) found to be 8. The corresponding metrics are 0.714, 0.969, 0.182, and 0.166, respectively. The most effective CoMSIA model produced a q^2^ statistic of 0.705, an impressive r^2^ value of 0.865, a MAE value of 0.271, a SEE value of 0.336, and an ONC value of 3. Analyses conducted with CoMFA and CoMSIA produced q² and r² values exceeding 0.5 and 0.6, respectively, indicating acceptable predictive and explanatory power. The CoMSIA methodology produces five unique molecular force fields, each contributing at different levels. The distribution across the five force field terms comprises: steric force (S) (2.51%), electrostatic force (E) (43.43%), hydrophobic force (H) (5.93%), hydrogen bond acceptor (A) (3.51%), and hydrogen bond donor (D) (44.56%). By systematically organizing and integrating all five molecular force fields, a total of 31 model sets were generated. As shown in Table S3, all 31 possible CoMSIA combination descriptors were examined and documented. As shown in Fig. 4, the hydrophobic and steric (HS) fields yield the maximum q² value (0.72), implying that q² emerges when hydrophobic and steric descriptors are employed together with other fields.

### External validation of CoMFA and CoMSIA methods

To enhance the assessment of the predictive capability and dependability of the CoMFA and CoMSIA models, an external validation was conducted. Initially, the CoMFA and CoMSIA models were validated with a test set to assess predictive performance. The calculated $\:{r}_{test}^{2}$ values were 0.871 for CoMFA and 0.879 for CoMSIA. Although the CoMFA model showed strong performance on the training set (r² = 0.969), its performance on the test set did not match this level ($\:{\mathrm{r}}_{\mathrm{t}\mathrm{e}\mathrm{s}\mathrm{t}}^{2}$ = 0.871). In contrast, the r^2^ values of the test and training phases in the CoMSIA model demonstrate a reasonably strong alignment.

Moreover, the external predictive capability of the established 3D-QSAR models was evaluated using the test set, taking into account the $\:{r}_{m}^{2}$ metrics as illustrated below:

$$\:{\mathrm{r}}_{\mathrm{m}}^{2}={\mathrm{r}}^{2}(1-\sqrt{\left|{\mathrm{r}}^{2}-{\mathrm{r}}_{0}^{2}\right|}\:)$$

Within this equation, r² denotes the squared correlation coefficient, which assesses the concordance between predicted and observed activity levels over the entire compound set. $\:{\mathrm{r}}_{0}^{2}$ is calculated as follows:

$$\:{\mathrm{r}}_{0}^{2}=1-\frac{\sum\:{({\mathrm{y}}_{\mathrm{i}}-{\mathrm{y}}_{\mathrm{i}}^{\mathrm{r}0})}^{2}}{\sum\:{({\mathrm{y}}_{\mathrm{i}}-{\stackrel{-}{\mathrm{y}}}_{\mathrm{i}})}^{2}}$$

$$\:{\mathrm{y}}_{\mathrm{i}}^{\mathrm{r}0}={\mathrm{k}\mathrm{y}}_{\mathrm{i}}$$

and

$$\:\mathrm{k}=\frac{\sum\:{\mathrm{y}\mathrm{y}}_{\mathrm{i}}}{\sum\:{\mathrm{y}}_{\mathrm{i}}^{2}}$$

where y_i_ is the predicted activity of the test set of compounds, and y is the experimental activity of the test set of compounds, $\bar{\mathrm{y}}$ represents the average value of compound activity in the test set. A summary of the external validation parameters for CoMFA and CoMSIA has been presented in Table 1.

As described by Golbraikh and Tropsha, the 3D-QSAR model developed in this study underwent comprehensive validation, employing multiple evaluation metrics, including$\:\:{\mathrm{r}}_{\mathrm{t}\mathrm{e}\mathrm{s}\mathrm{t}}^{2}{,\:\:\mathrm{r}}_{0}^{2}$, and $\:{\:\mathrm{r}}_{\mathrm{m}}^{2}$. Collectively, these performance indicators attest to the model’s dependable behavior and its overall predictive accuracy in the examined contexts^40^.

**Table 1:** Summary of external validation parameters for CoMFA and CoMSIA.

Parameters	Threshold value	CoMFA	CoMSIA
$\:{\mathrm{r}}_{\mathrm{t}\mathrm{e}\mathrm{s}\mathrm{t}}^{2}$	> 0.6	0.871	0.879
k	$\:0.85\le\:\mathrm{k}\:\le\:1.15$	0.99	0.998
$\:{\mathrm{r}}_{0}^{2}$	Close to the value of $\:{\mathrm{r}}_{\mathrm{t}\mathrm{e}\mathrm{s}\mathrm{t}}^{2}$	0.948	0.951
$\:\frac{\left|\right({\mathrm{r}}_{\mathrm{t}\mathrm{e}\mathrm{s}\mathrm{t}}^{2}-{\mathbf{r}}_{0}^{2}\left)\right|}{{\mathrm{r}}_{\mathrm{t}\mathrm{e}\mathrm{s}\mathrm{t}}^{2}}$	$\:\frac{\left|\right({\mathrm{r}}_{\mathrm{t}\mathrm{e}\mathrm{s}\mathrm{t}}^{2}-{\mathrm{r}}_{0}^{2}\left)\right|}{{\mathrm{r}}_{\mathrm{t}\mathrm{e}\mathrm{s}\mathrm{t}}^{2}}<\:0.1$	0.088	0.082
$\:{\mathrm{r}}_{\mathrm{m}}^{2}$	$\:{\mathrm{r}}_{\mathrm{m}}^{2}>0.5$	0.629	0.643

### The applicability domain and Y-randomization test results

**Fig. 5:**
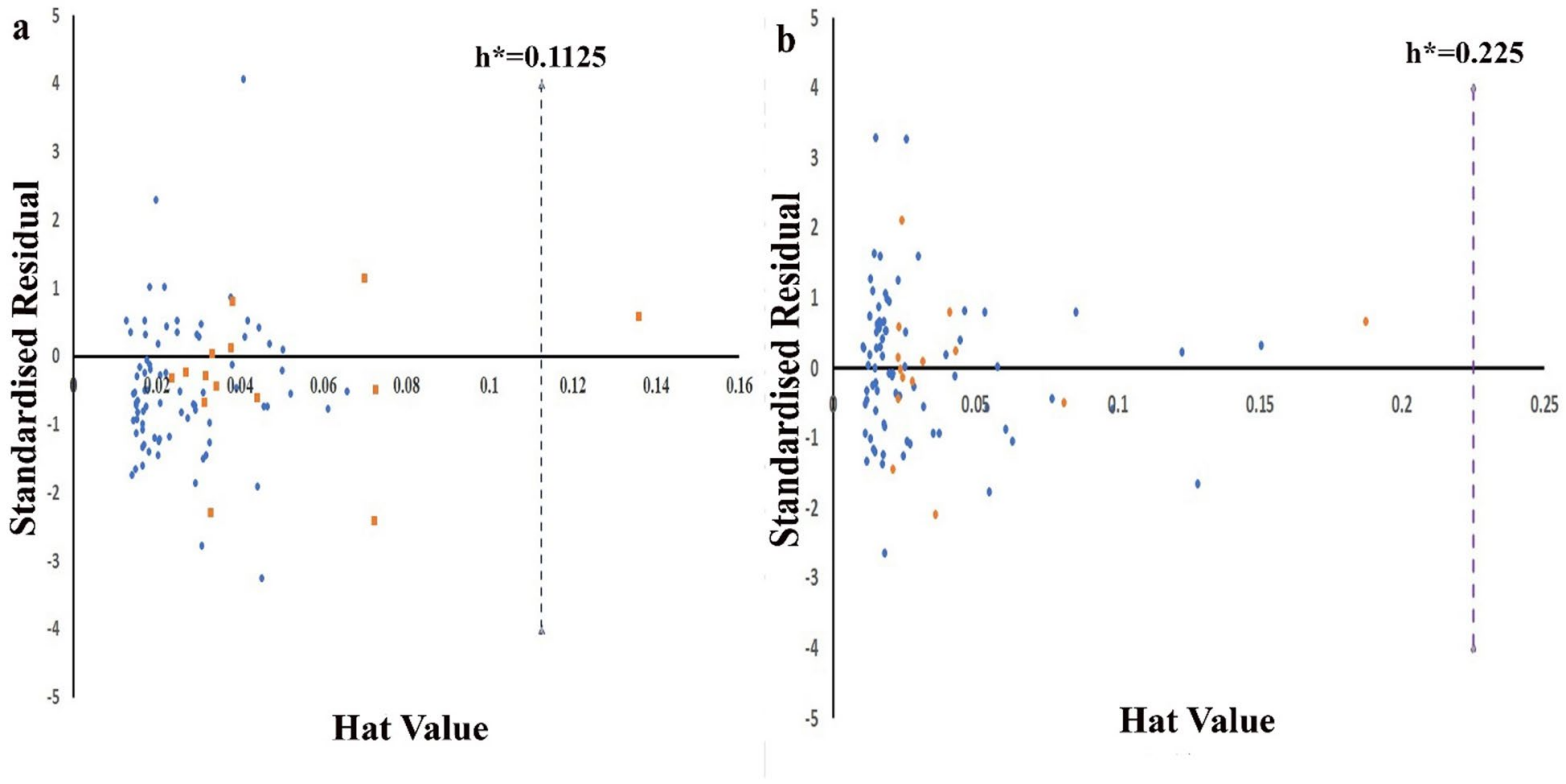
The applicability domain detection. (**a**) CoMFA model, (**b**) CoMSIA model (training compounds blue and test compounds red are presented).

The applicability-domain outcomes for the CoMFA and CoMSIA models are illustrated in Fig. 5. In the CoMFA model, all compounds in both the test and training sets exhibit good leverage, except for two training-category combinations that are response outliers (The standard residual is outside the range of ± 3), and a single test-category combination that is a bad leverage. In the CoMSIA model, all compounds from the test and training sets show good leverage, aside from two training combinations identified as response outliers.

Training and test compounds, in both CoMFA and CoMSIA models, are in the leverage range, implying a broad applicability domain and the capacity to predict activities of new compounds. One test-set compound in the CoMFA model, however, exceeds the threshold, necessitating a careful analysis of its attributes and contributions to safeguard the model’s reliability under specific scenarios.

Table 2 presents the Y-randomization test results for the CoMFA and CoMSIA models, where all observed r² values are low, indicating that the model’s performance cannot be attributed to random correlations in the training set.

**Table 2:** Y-randomization test result for CoMFA and CoMSIA models.

Model	*r*^2^ CoMFA	*r*^2^ CoMSIA
Orginal	0.871	0.879
1	0.133	0.015
2	0.003	0.265
3	0.146	0.186
4	0.011	0.156
5	0.243	0.209
6	0.140	0.032
7	0.037	0.282
8	0.055	0.256
9	0.251	0.045
10	0.088	0.056

### CoMFA and CoMSIA contour maps

**Fig. 6:**
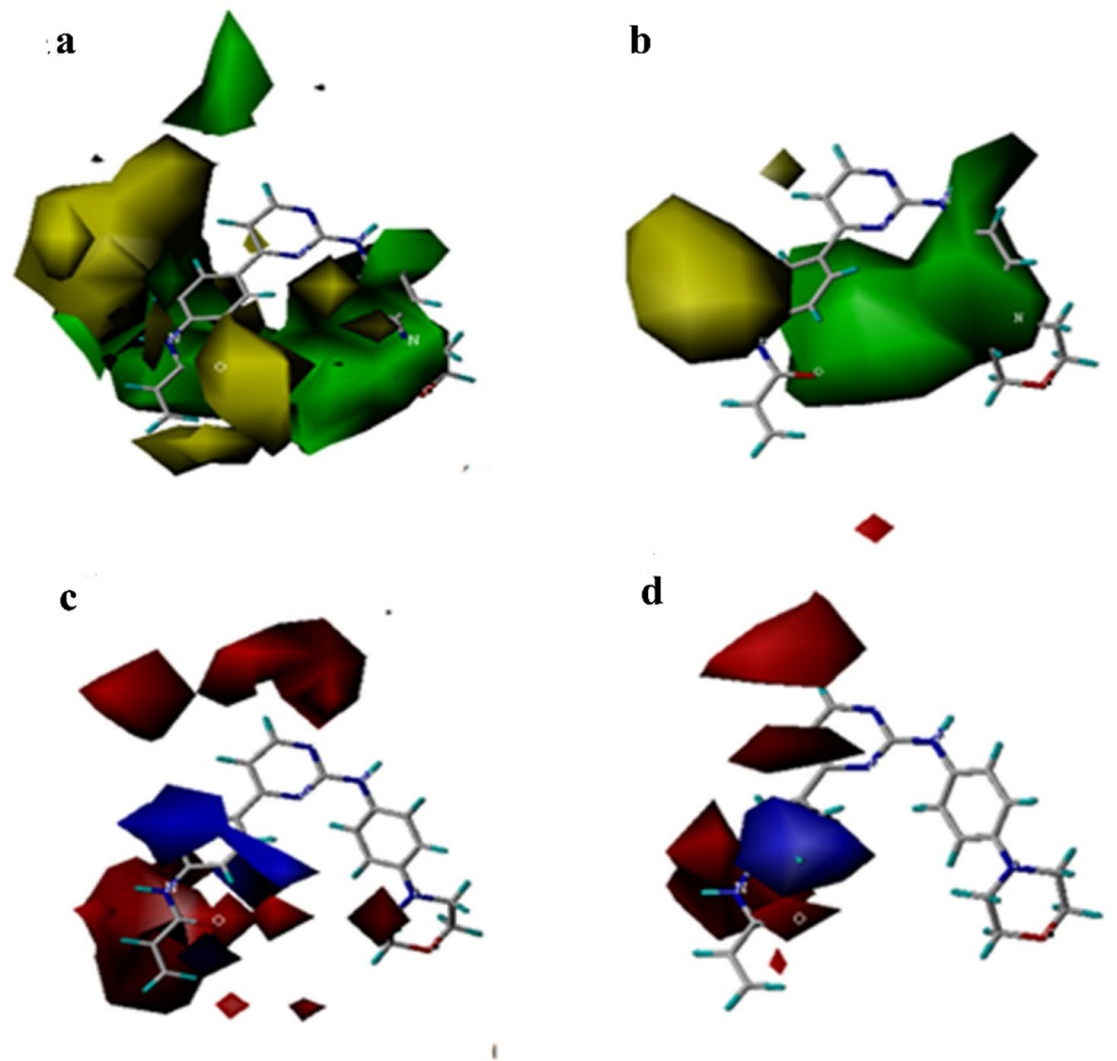
Steric and electrostatic CoMFA and CoMSIA contour maps of compound 12(A). (**a**) CoMFA steric map, (**b**) CoMSIA steric map; favored regions shown in green and disfavored regions in yellow, (**c**) CoMFA electrostatic map, and (**d**) CoMSIA electrostatic map; highlighting electropositive regions in blue and electronegative regions in red.

**Fig. 7:**
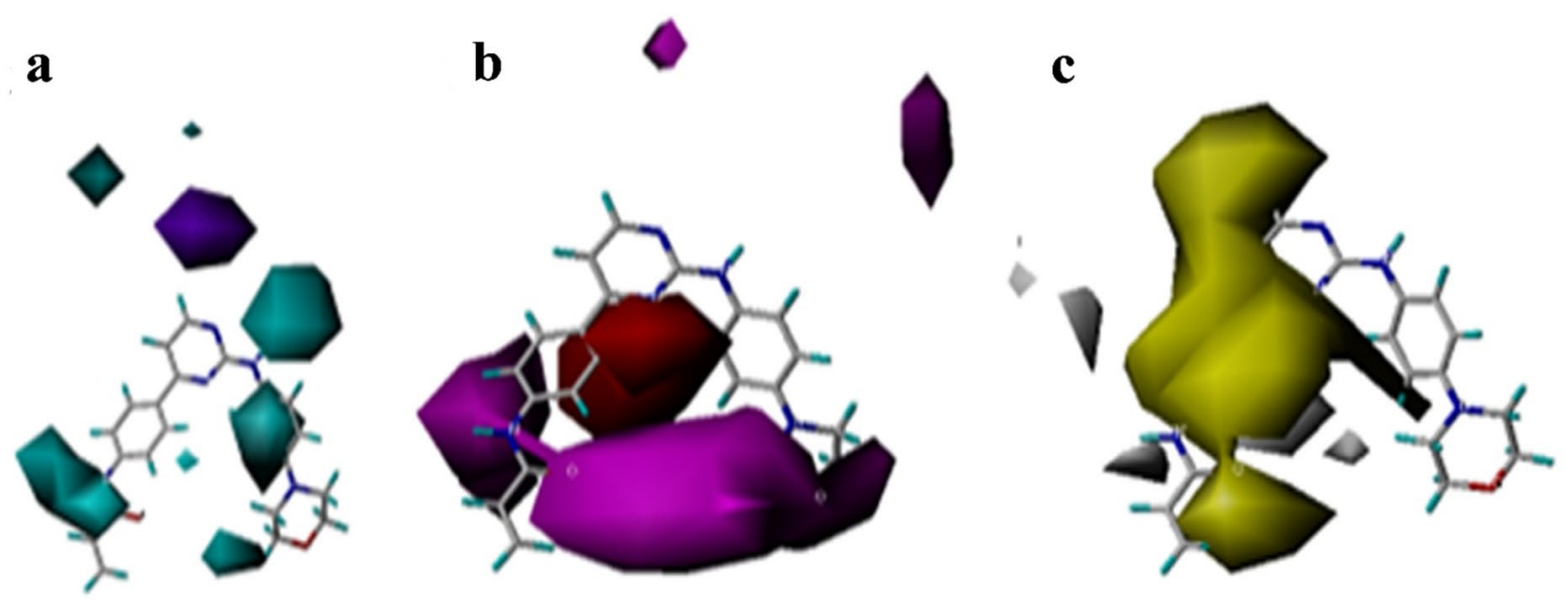
Hydrogen bond donor, Hydrogen bond acceptor, and hydrophobic CoMSIA contour maps of compound 12(A). (**a**) The hydrogen bond donor map, with favored areas in cyan and less favorable ones in purple; (**b**) the hydrogen bond acceptor map, magenta favored and red for less favorable areas; (**c**) the hydrophobic map, favored regions in yellow and less favorable regions in white.

The contour plots produced by the CoMFA and CoMSIA analyses offer meaningful insights into how a compound’s structural features relate to its pharmacological activity (Figs. 6 and 7). The study involved the creation of multiple contour plots in CoMFA and CoMSIA, representing the electrostatic field, molecular steric field, hydrogen bond acceptor field, hydrogen bond donor field, and hydrophobic field utilizing the StDev*Coeff method. By analyzing the color variations across distinct regions in the contour plots, the relationship between the compound’s structural features and its pharmacological activity was clarified. The reference structure employed in the contour plots for both CoMFA and CoMSIA analyses was Compound 12(A).

The steric field of CoMFA and CoMSIA is illustrated in Fig. 6a,b, respectively. The green contour delineates areas where the presence of bulky substituents would enhance the inhibitory activity of JAK3; conversely, the yellow contour signifies regions where steric bulk would diminish the inhibitory effect. Green contours surrounding the phenyl rings and morpholine ring, particularly in the inner regions of the 3D structure, indicate that these areas are conducive to steric interactions. Conversely, the yellow contour in the outer regions near the phenyl ring and amide group suggests that smaller groups should be positioned here due to the presence of unfavorable steric interactions.

The blue contour delineates the favorable positions for electropositive groups, while the red contour indicates the regions that favor electronegative groups (Fig. 6c,d). In the CoMFA and CoMSIA contour maps, concerning the electrostatic contour maps, the red polyhedra surrounding the pyrimidine ring and amide group represent the primary locations that should provide electronegative characteristics in this area. In proximity to the phenyl ring (adjacent to the amide group), the blue polyhedron suggests that an electropositive center is advantageous for activity.

The hydrogen bond donor (HBD) map for the CoMSIA model is illustrated in Fig. 7a. In the CoMSIA framework, regions that favor hydrogen bond donors are depicted with cyan contours, while regions that are unfavorable are indicated by purple contours. The presence of a favorable cyan contour near the NH groups and the phenyl ring associated with the morpholine moiety suggests that hydrogen bond donor groups in these regions enhance JAK3 inhibitory activity. In contrast, an unfavorable purple contour in the outer region of the pyrimidine ring indicates a diminished or non-beneficial contribution of HBD in that area.

In the contours of hydrogen bond acceptors (HBA) (Fig. 7b), the magenta contour highlights regions that are favorable for hydrogen bond acceptor substituents on ligands. In contrast, the red contour indicates areas where these substituents and compounds are likely to be less favored. The prominent magenta outline encircling the amide portion and the oxygen atom of the morpholine ring signifies areas that are preferred for hydrogen bond acceptors. The red outline enveloping the pyrimidine and phenyl rings emphasizes a region that is unfavorable for HBA.

Hydrophobic contour maps for the CoMSIA model are shown in Fig. 7c. The yellow regions indicate zones where hydrophobic character is favored, while the grey areas denote zones where hydrophilic character is favored. A substantial hydrophobic contour, highlighted in yellow, was identified proximal to the phenyl ring and the carbonyl moiety of the amide. In contrast, the hydrophilic group is seen near the nitrogen atom of the amide.

### Design of new compounds

According to the contour maps derived from CoMFA and CoMSIA illustrated in Figs. 6 and 7 and a total of 103 novel inhibitors have been suggested. As illustrated in Fig. 7, the importance of the NH group within the amide moiety for HBD interactions, coupled with the function of the carbonyl group as an HBA, resulted in the choice to maintain the amide chain in its initial position throughout all newly designed structures. Fusion of pyrrole and thiophene rings, along with cyclopentene and cyclohexene dienes, to the amide-bearing phenyl ring can enhance steric effects. The thiophene ring, cyclopentene, and cyclohexene dienes play a crucial role in enhancing both steric and hydrophobic interactions. Moreover, the addition of smaller groups to these rings, such as methyl ether, alkyl groups like methyl, ethyl, and halogens like F and Cl, enhances hydrophobic interactions. The pyrimidine moiety was consistently retained in all newly designed structures; in certain instances, it was connected to a minor amine or alcohol group to enhance electronegative interactions. The benzene ring linked to the morpholine ring was preserved in all the new structures, with some modifications that included an alkyl alcohol group to improve hydrogen bond donation. In numerous new designs, the morpholine ring was replaced with a medium-length chain that had a hydroxyl (OH) or NH group at its terminal position to increase both hydrogen bond donation and hydrogen bond acceptance, or with a chain that incorporated either an ester or ether group to enhance hydrogen bond acceptance.

### Molecular docking and MM-GBSA analysis

The initial step was carried out to validate the docking process for the JAK3 protein alongside the crystallographic ligand (N-(3-{[(5-chloro-2-{[2-methoxy-4-(4-methylpiperazin-1-yl)gphenyl]amino}pyrimidin-4-yl)amino]methyl}phenyl)prop-2-enamide).

**Fig. 8:**
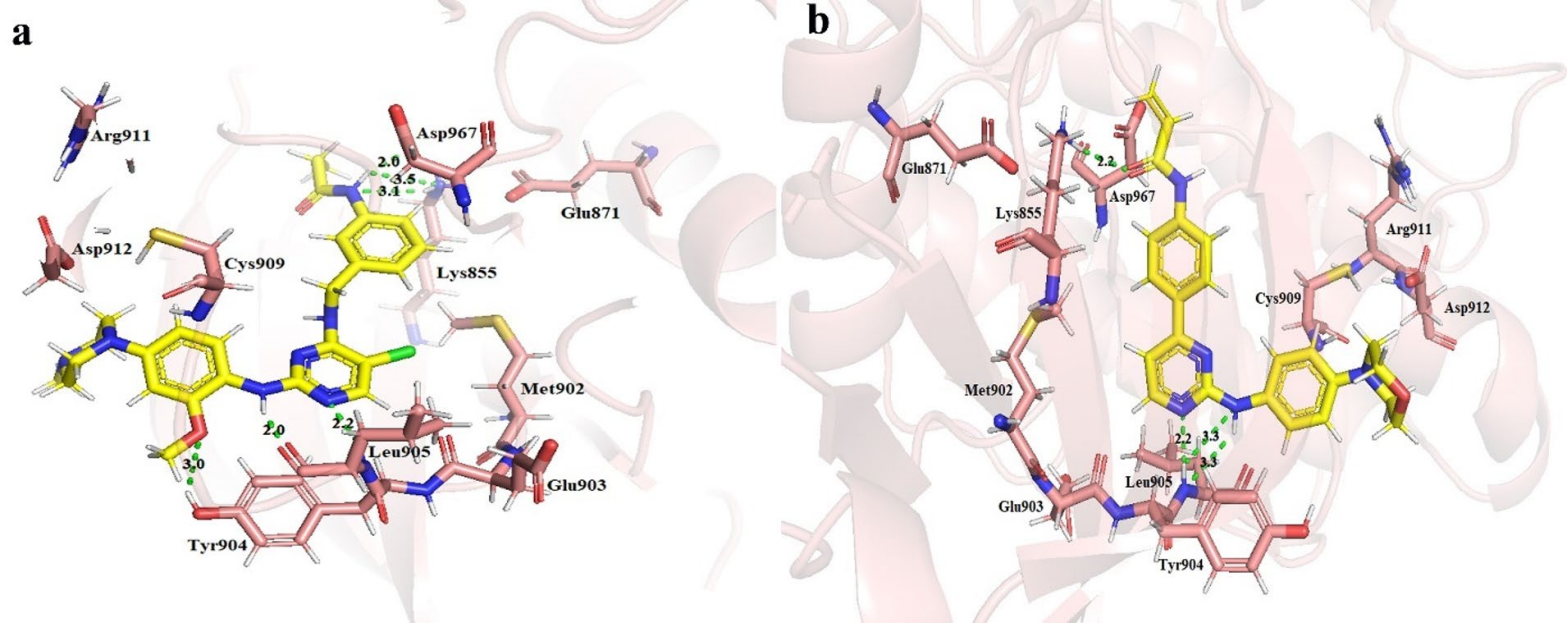
(**a**) 3D structure of the crystallographic ligand. (**b**) 3D structure of compound 12(A) as a template.

The JAK3 binding pocket is defined by a mixture of hydrophobic and hydrophilic components, as well as structural water molecules. Key residues found in this pocket include Lys855, Glu871, Met902, Glu903, Cys909, Arg911, Asp912, and Asp967, with hydrogen bonds observed with the amino acids Tyr904 and Leu905^15^. Subsequently, docking studies were conducted on compound 12(A) as the template. The calculated XP-GScore for the crystallographic ligand and compound 12(A) within the JAK3 active site is -9.549 kcal/mol and − 9.804 kcal/mol, respectively. The main interactions are shown in Fig. 8.

The Docking technique is an important computational approach used for the identification of new JAK3 inhibitors, typically applied when structural data regarding the target receptor is accessible. It aids in forecasting energetically advantageous binding orientations of ligands to the target protein, thereby allowing for the prioritization of compounds according to their potential for effective interactions with the target. In this study, molecular docking was conducted on all the designed compounds utilizing Schrodinger 2015. The XP-GScore for the designed compounds ranges from − 1.725 to -14.162 kcal/mol. The eight compounds exhibiting the lowest XP-GScore values are detailed in Table S4. The best compounds involved the establishment of hydrogen bonds with significant residues, such as Leu905, Lys855, Cys909, Asp912, Tyr904, Glu903, and Asp967.

Accurate binding affinity predictions for the top-ranked hits are crucial in the virtual screening workflow to inform compound prioritization. The solvation energy terms of both the protein and ligand are critical thermodynamic factors in estimating free binding energy that are ignored by various docking tools. Improved methodologies, including MM-GBSA, merge molecular-mechanics force fields with continuum solvation models to produce more reliable predictions of ligand binding free energy^41^.

**Fig. 9:**
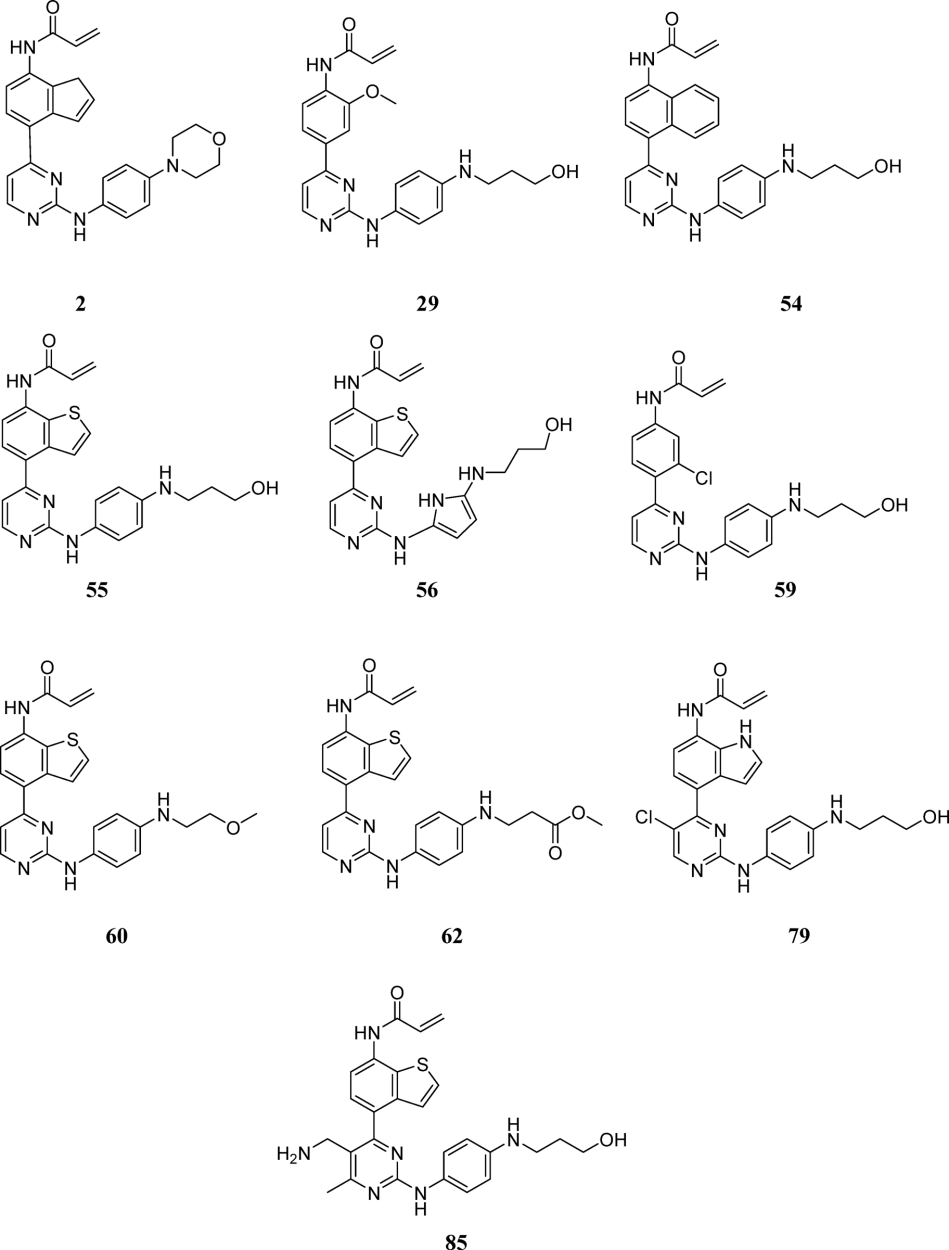
Chemical structure of ten obtained molecules using the MM-GBSA method.

The results indicated that ten molecules demonstrated lower free binding energies compared to the 12 (A) ligand (-78.908 kcal/mol) (Fig. 9). As illustrated in Table 3, compound 56 exhibited the most negative binding energy, recorded at -102.888 kcal/mol. The increased binding energy observed for compound 56 correlated with higher van der Waals interaction energy (-54.955 kcal/mol) and lipophilic interaction energy (-46.131 kcal/mol). This indicates that the compound demonstrated greater stability within the protein binding site relative to the others, thereby exhibiting the strongest in silico binding affinity for the JAK3 pocket. Among three other selected compounds, compound 2 demonstrated a binding energy of -97.643 kcal/mol, which is relatively close to that of the optimal compound, 56. Table 2’s free-energy components analysis shows that ΔG_vdW_ is the principal contributor to ligand binding, with a value of − 51.335 kcal/mol. This outcome highlights the important role of London dispersion and dipole–dipole interactions in stabilizing the ligand–protein complexes, a conclusion that is consistent with the involvement of hydrophilic amino acids in the binding site. Furthermore, in compound 2, the significance of lipophilic energy (-45.755 kcal/mol) is evident when compared to other interactions, attributable to the existence of hydrophobic amino acids within the active site. The top five molecules (56, 2, 55, 79, and 85) with the lowest binding energies were selected for further studies.

**Table 3:** MM/GBSA results of template and predicted compounds.

Comp.	∆G_Binding_ (kcal/mol)	∆G_Coulomb_ (kcal/mol)	∆G_Covalent_ (kcal/mol)	∆G_Hbond_ (kcal/mol)	∆G_Lipo_ (kcal/mol)	∆G_vdW_ (kcal/mol)
**12(A)**	− 76.908	− 18.683	3.216	− 1.615	− 33.276	− 47.334
**79**	− 95.861	− 31.664	5.417	− 2.768	− 45.126	− 48.498
**55**	− 97.440	− 35.978	5.304	− 2.254	− 43.013	− 48.222
**54**	− 94.922	− 36.244	6.541	− 2.244	− 40.041	− 49.297
**62**	− 94.619	− 30.443	6.622	− 1.513	− 41.790	− 54.902
**9**	− 93.655	− 36.156	2.681	− 2.318	− 36.539	− 46.768
**59**	− 94.632	− 32.716	0.861	− 2.234	− 40.747	− 44.789
**56**	− 102.888	− 25.217	1.818	− 3.148	− 46.131	− 54.955
**60**	− 93.278	− 21.396	4.232	− 1.434	− 43.322	− 54.492
**2**	− 97.643	− 20.957	1.559	− 1.261	− 45.755	− 51.335
**85**	− 95.878	− 19.866	− 1.200	− 1.419	− 46.679	− 54.901

### Molecular dynamics simulation analysis

Molecular Dynamics (MD) simulation is a fundamental in silico approach for characterizing ligand-induced conformational changes and fluctuations in protein structure over time. Unlike molecular docking, MD takes into account the intrinsic flexibility and dynamic behavior of macromolecules, providing a more biologically relevant representation of systems under cellular physiological conditions. Consequently, the best ligand–protein complexes were simulated for 100 ns in a fully hydrated environment. The simulations aimed to probe the persistence of the proposed ligand orientations within the JAK3 binding site during the trajectory, with the backbone RMSD of the protein tracked over time to gauge stability for compounds 56, 2, 55, 79, and 85 in complex with JAK3.

**Fig. 10:**
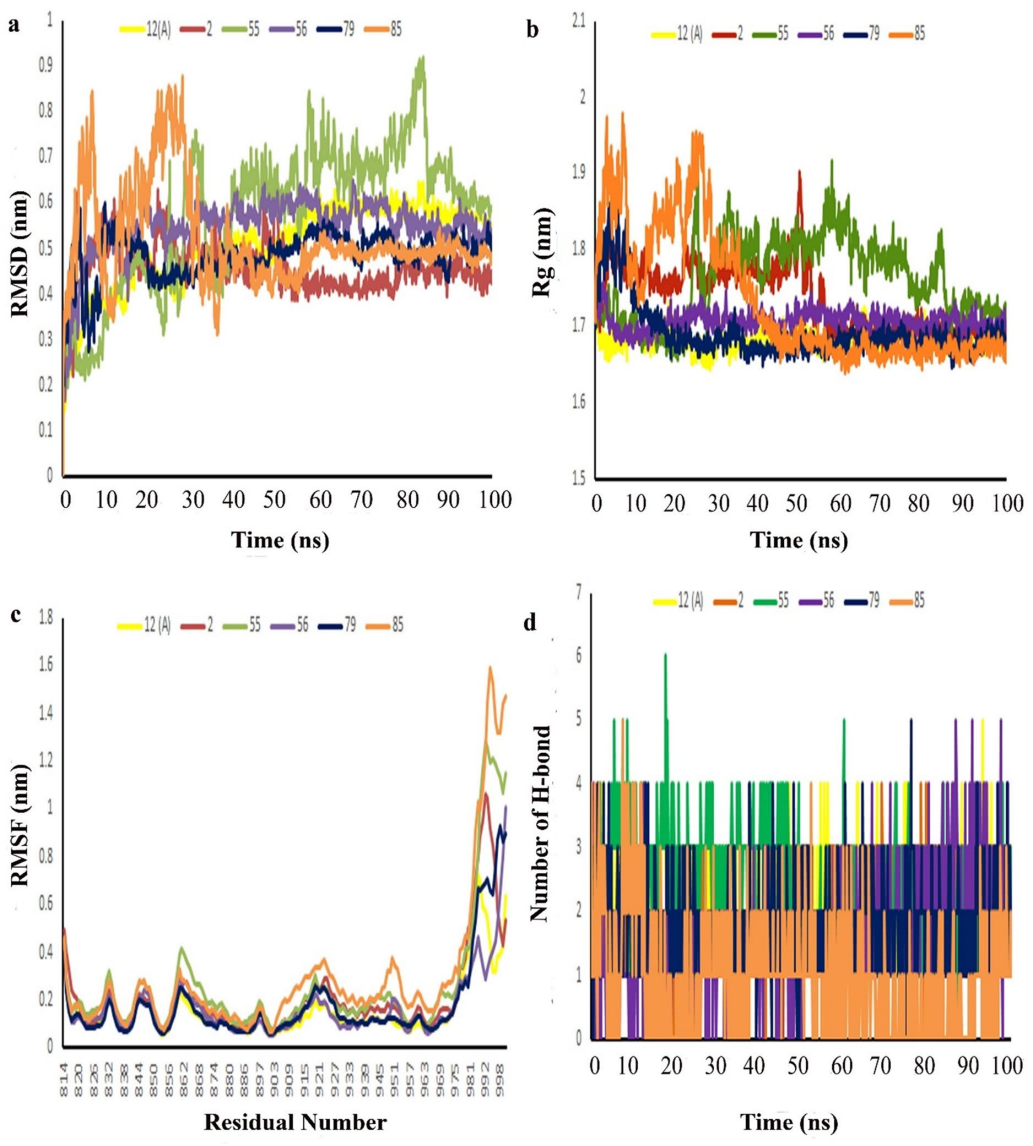
Molecular dynamics simulations. (**a**) RMSD plot measured in nanometers during 100 ns. (**b**) Rg plot measured in nanometers during 100 ns. (**c**) RMSF plot measured in nanometers for each residue number. (**d**) Number of hydrogen binding during 100 ns. Compound 2 is represented in red, compound 79 in navy blue, compound 55 in green, compound 56 in purple, compound 85 in orange, and the reference compound (12 A) in yellow.

The backbone RMSD of the protein is expected to stay below 3 Å throughout the equilibration phase. A higher RMSD value indicates a greater degree of conformational changes that the system has undergone. As shown in Fig. 10a, the relatively flat RMSD slope observed during the simulation period for compounds 79, 56, and 2 serves as a strong indicator of a more stable system, similar to that of 12(A). The backbone of the complex in compound 79 showed fluctuations during the first 10 ns, spanning RMSD values of 0.23–0.59 nm, with an average around 0.49 nm until stabilization. In compound 56, backbone dynamics were observed in the first 20 ns (RMSD 0.16–0.59 nm), with an ensuing average fluctuation of ~ 0.57 nm leading to stabilization. The backbone of the complex in compound 2 showed fluctuations in the first 24 ns, spanning RMSD 0.23–0.59 nm, then equilibrated with an average of ~ 0.48 nm. Compound 85 exhibited stabilization after 35 ns, with an average RMSD of approximately 0.52 nm. In contrast, compound 55 shows pronounced fluctuations in the RMSD trajectory, indicating a less stable ligand–protein interaction relative to the other compounds.

The gyration radius (Rg), an indicator of protein compactness, was assessed. A reduced Rg reflects a tighter, more compact arrangement (spherical or densely packed), while an elevated Rg indicates an extended, looser conformation. Figure 10b shows that, for nearly all compounds, the Rg trajectories align with compound 12(A); except compound 85, which aligns after 40 ns until the conclusion of the simulation. A persistent pattern of stable curves and diminished Rg values is evident throughout the entire simulation period, indicating a strong binding affinity among the complex’s components. Nonetheless, variations in Rg values are observed, stemming from the dynamic ligand–protein interactions that cause changes in the protein’s internal environment. Particularly, the ligand–protein complexes can adjust to the new environment and exhibit promising mutual interactions, as demonstrated.

RMSF assessments provide a comprehensive picture of how individual residues in the protein backbone move, considering their locations and roles in ligand binding. This approach facilitates the detection of flexible regions within the protein. Elevated RMSF values indicate larger atomic deviations from mean positions during the MD run. In the RMSF context shown in Fig. 10c, the fluctuation profiles of the studied complexes are similar to those observed for JAK3 bound to 12(A). For the entire set of complexes, RMSF values spanned roughly 0.07–0.34 nm, reflecting robust protein stability and limited conformational changes. Across these complexes, the binding-site residues Lys855, Glu871, Met902, Glu903, Tyr904, Leu905, Cys909, Arg911, Asp912, and Asp967 exhibited minimal fluctuations during the MD runs, averaging about 0.1 nm. This limited mobility supports the notion that these residues can maintain stable ligand interactions within the binding site.

Hydrogen-bond formation was thoroughly examined along the MD trajectories. Figure 10d shows that the H-bond count varied from 0 to 5 for the 12(A)–JAK3 complex and for the JAK3 complexes with 56, 79, and 85. Among the evaluated complexes, the H-bond pattern of 56-JAK3 most closely matched that of 12(A)-JAK3. Compound 55-JAK3 demonstrated the highest number of H-bonds, ranging from 0 to 6, whereas compound 2-JAK3 exhibited the lowest, with a range of 0 to 4.

### Free-energy landscape

**Fig. 11:**
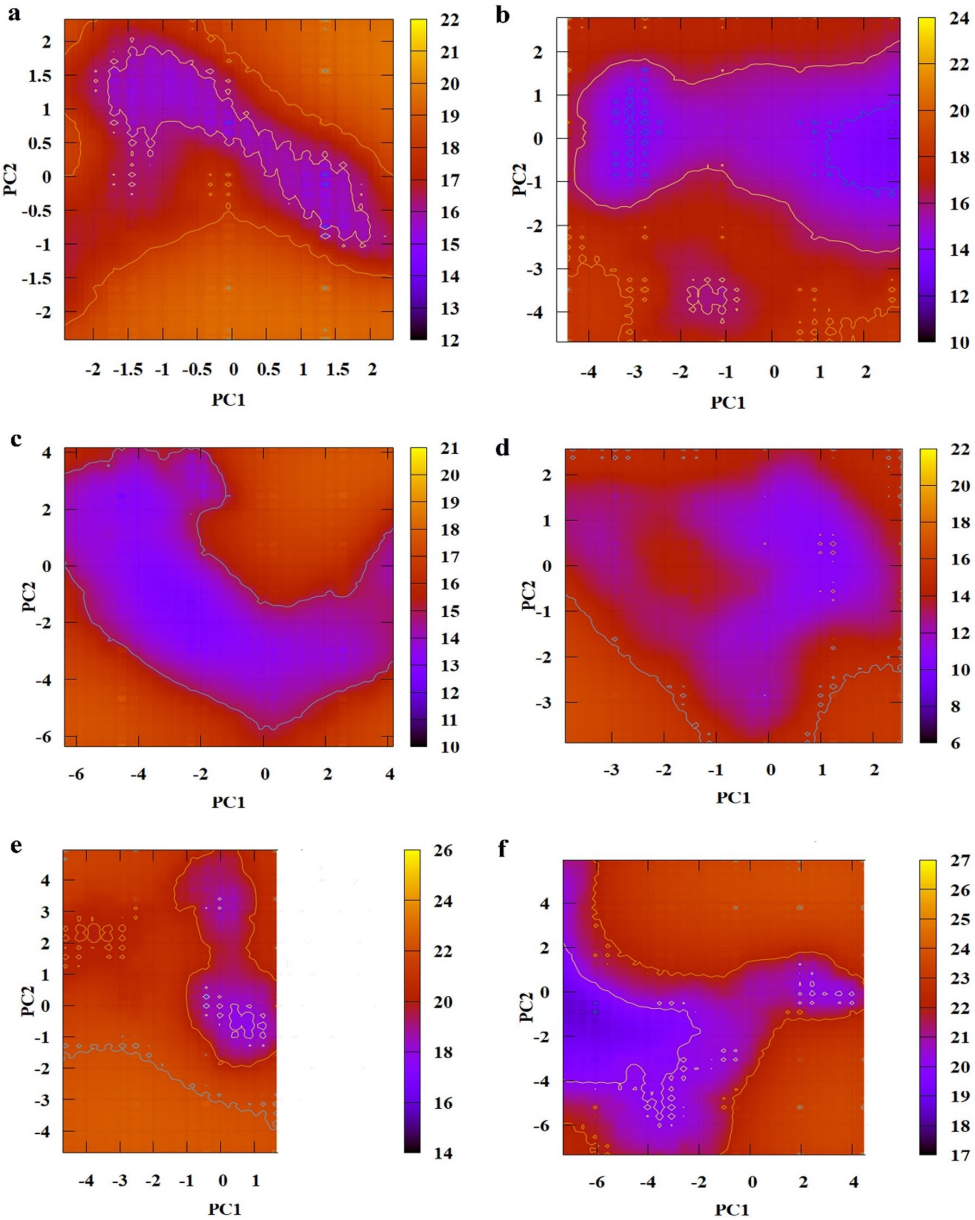
2D PCA-derived free energy landscape plots for the protein–ligand complexes sampled during the 100 ns MD trajectory. (**a**) JAK3-12(A) complex, (**b**) JAK3-2 complex, (**c**) JAK3-55 complex, (**d**) JAK3-56 complex, (**e**) JAK3-79 complex, and (**f**) JAK3-85 complex.

**Fig. 12:**
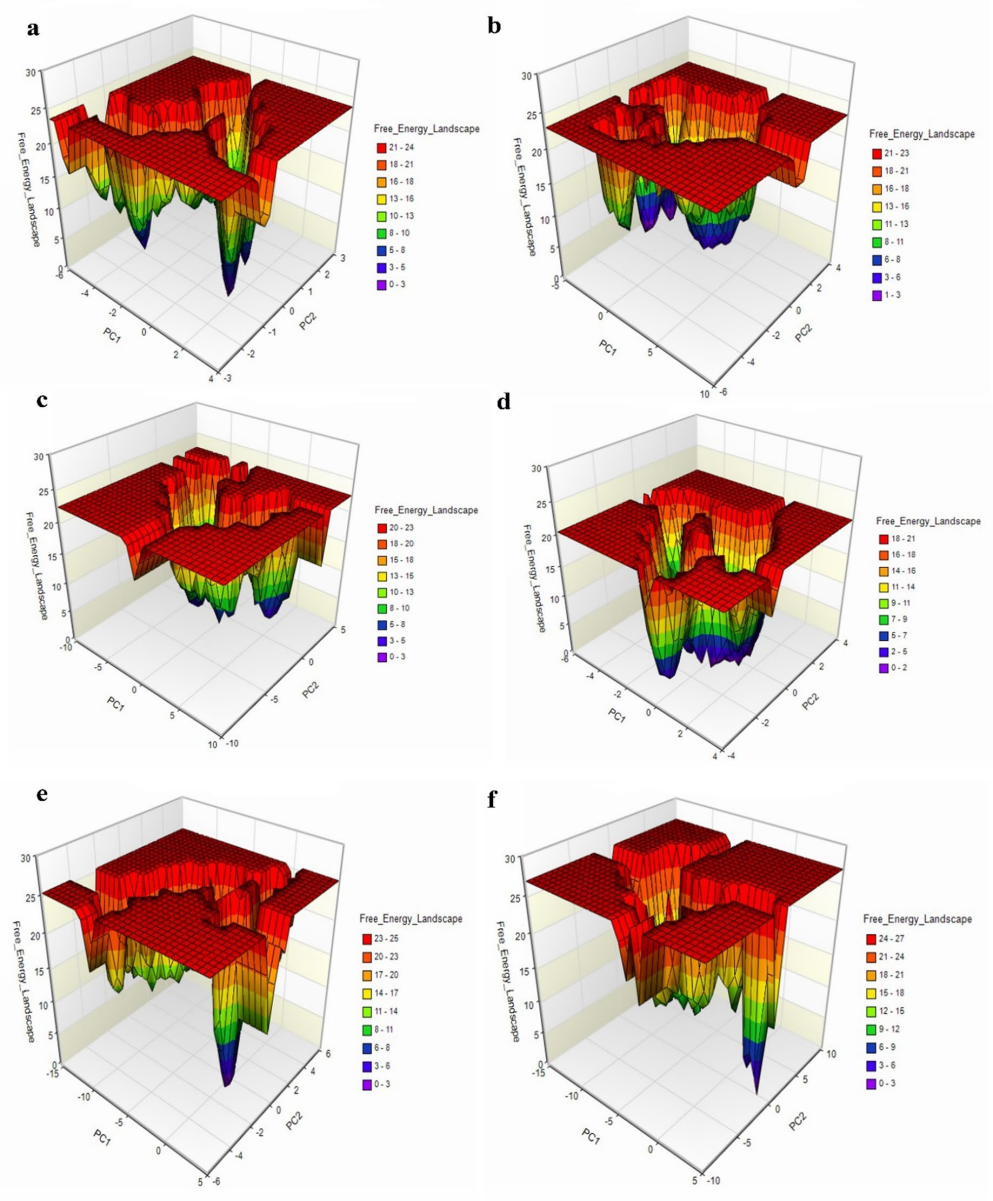
3D PCA-derived free energy landscape plots for the protein–ligand complexes sampled during the 100 ns MD run. (**a**) JAK3-12(A) complex, (**b**) JAK3-2 complex, (**c**) JAK3-55 complex, (**d**) JAK3-56 complex, (**e**) JAK3-79 complex, and (**f**) JAK3-85 complex.

To illuminate the energy distribution along the MD-sampled protein folding pathway, the FEL was projected onto the principal component. A two-dimensional FEL map for the protein–ligand complexes was built from PCA-derived eigenvector coordinates. In addition, a FEL contour map with distinct color gradations (yellow, orange, red, and blue) was produced to evaluate the maximum and minimum energy levels of the ligand–JAK3 complexes. The complexes of JAK3-designed compounds exhibit a greater number of stable conformations in comparison to JAK3-12(A), as illustrated in Fig. 11. Furthermore, the Gibbs free energy for JAK3-12(A) was observed to be within the ranges of 12 to 22 kJ/mol, and JAK3-56 complexes, as the best complex was observed to be within the ranges of 6 to 22 kJ/mol. Moreover, three-dimensional plots of the free energy for the JAK3-12(A) complex and for the JAK3–designed compound complexes are presented in Fig. 12. The compounds 2 and 56 showed the most prominent and stable conformations (Fig. 12(b) and (d)) compared to other complexes in Fig. 12. This may indicate that compounds 2 and 56 can provide a stable binding at the JAK3 active site of the protein.

### Dynamic cross-correlation map (DCCM)

**Fig. 13:**
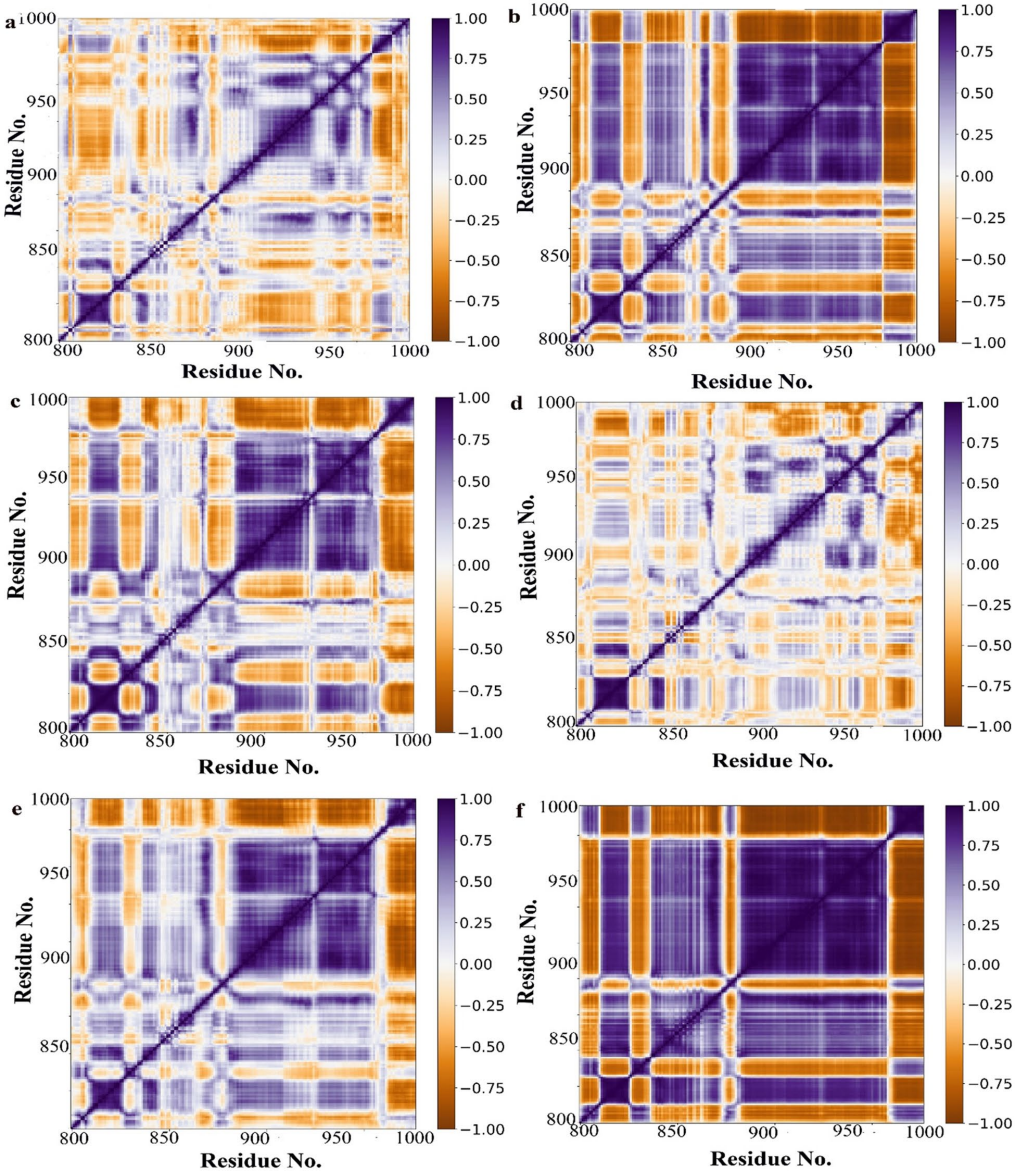
The correlation matrix heatmap of JAK3 residues for the MD simulation study. (**a**) JAK3-12(A) complex, (**b**) JAK3-2 complex, (**c**) JAK3-55 complex, (**d**) JAK3-56 complex, (**e**) JAK3-79 complex, and (**f**) JAK3-85 complex.

The DCCM analysis, performed for each simulation study, focused on the C-alpha chain of JAK3 and is presented in Fig. 13. In the DCCM map, the purple regions denote high positive correlation, while the orange regions denote strong negative correlation. The color gradient between them reflects intermediate correlation values. The diagonal elements represent self-correlation (*r* = 1.0), appearing as the strongest positive signal^42^. The correlation matrix in Fig. 13 shows that JAK3 residues did not move similarly across all simulated complexes (Fig. 13a–f). The DCCM indicates analogous co-movements of the active-site residues in the JAK3–12(A) and JAK3–56 complexes (Fig. 13a and d), while correlations for the other compounds are minimal. For JAK3 bound to compound 2 and 85, the DCCM demonstrates similar coupled motions among active-site residues (Fig. 13b and f), with substantially lower correlation in the other ligand complexes.

### Probability density function (PDF) analysis

To assess the impact of the ligand on the stability of JAK3 configurations, the protein-density function obtained from 100 ns molecular dynamics trajectories for six complexes (a–f) was compared against the reference complex a, 12 (A), which functioned as a control (Figure S2). The results show that the peaks for all complexes remain at the longitudinal center of the system (≈ 2.8–3.0 nm), while the center of stability and peak width exhibit slight deviations from complex 12(A). Complexes characterized by narrower peaks demonstrate enhanced configurational stability and a more limited range of motion; conversely, larger peak widths imply an expanded range of motion or the presence of multiple, distinct configurations^43^. The shifts in peak position or width observed for complexes b–f relative to complex a reflect the ligand’s impact on the protein’s internal connectivity network and the dynamics of the active site.

## Conclusion

Rheumatoid arthritis is a chronic autoimmune disease characterized by joint pain and inflammation. Janus kinase inhibition is an established therapeutic strategy for RA, but first‑generation non‑selective JAK inhibitors, despite their efficacy, are associated with adverse events due to broad cytokine inhibition. Therefore, achieving selectivity toward JAK3 remains an important goal in RA therapy. Several structural classes, including pyrrolopyrimidines, pyrrolopyridines, pyrimidines, and pyrazolopyrimidines, have been explored as JAK3 inhibitors; however, none have yet reached the pharmaceutical market.

In the present study, a parallel ligand-based pharmacophore (3D-QSAR) study and structure-based modeling/MD simulations were conducted to pinpoint new JAK3 inhibitors. The pharmacophore-guided alignment of the pyrimidine ring supported a robust 3D-QSAR model, identifying key 3D features that affect activity. Within the contour maps, HS (hydrophobic and steric) fields were the most predictive, represented by two CoMFA maps and five CoMSIA map analyses. Based on the 3D-QSAR model, 103 compounds were designed. The binding interactions of these candidates at the JAK3 active site were characterized by molecular docking and MM-GBSA. Ten compounds exhibited lower (more favorable) binding free energies than the reference compound 12(A). To assess the stability of the predicted ligands within the JAK3 active site, MD simulations were performed for the top five candidates (56, 2, 55, 79, and 85). Compound 56 emerged as the most promising candidate (ΔG_binding_ = -102.888 kcal/mol), showing superior binding stability along with the lowest RMSD fluctuations and radius of gyration (Rg). Moreover, the free energy landscape of the JAK3–56 complex indicated a greater number of stable conformations than JAK3–12(A). Also, the DCCM study findings confirm compound 56 as the closest analogue to reference compound 12(A). Structural analysis suggests that replacing the morpholine ring with an amino alkyl alcohol chain and the phenyl ring with a benzothiophene moiety may enhance JAK3 inhibition. Collectively, these computational results provide a solid basis for experimental validation, with subsequent in vitro and in vivo studies recommended to confirm the in silico inhibitory effects and to evaluate the pharmacological properties of these compounds.

## Supplementary Information

Below is the link to the electronic supplementary material.

Supplementary Material 1

## Data Availability

Data is available upon request from the corresponding author.

## Abbreviations

RA: Rheumatoid arthritis
NSAIDs: Non-steroidal anti-inflammatory drugs
DMARDs: Disease-modifying anti-rheumatic drugs
JAK: Janus Kinase
STAT: Signal transducers and activators of transcription
QSAR: Quantitative structure-activity relationship
3D-QSAR: Three-dimensional quantitative structure-activity relationship
MD: Molecular dynamics
CoMFA: Comparative molecular field analysis
CoMSIA: Comparative molecular similarity index analysis
IC_50_: Half-maximal inhibitory concentration
PLS: Partial least squares
PDB: Protein data bank
OPLS: Optimized potentials for liquid simulations
LigPrep: Ligand preparation
XP: Extra precision
MM/GBSA: Molecular mechanics with generalized Born and surface area solvation
MAE: Mean absolute error
q^2^: Quality of model
r^2^: Correlation coefficient
SEE: Standard error of the estimate
ONC: Optimal number of components
HBD: Hydrogen bond donor
HBA: Hydrogen bond acceptor
RMSD: Root-mean-square deviation
RMSF: Root-mean-square fluctuation
Rg: Gyration radius
FEL: Front-end loading
PCA: Principal component analysis

## References

[1] Smolen, J. S., Aletaha, D. & McInnes, I. B. Rheumatoid arthritis. *Lancet* **388** (10055), 2023–2038 (2016). 27156434 10.1016/S0140-6736(16)30173-8

[2] Jang, S., Kwon, E. J. & Lee, J. J. Rheumatoid arthritis: Pathogenic roles of diverse immune cells. *Int. J. Mol. Sci.* **23**(2). (2022). 10.3390/ijms23020905 PMC8780115 35055087

[3] Lin, Y. J., Anzaghe, M. & Schülke, S. Update on the pathomechanism, diagnosis, and treatment options for rheumatoid arthritis. *Cells* **9**(4). (2020). 10.3390/cells9040880 PMC7226834 32260219

[4] Huang, J. et al. Promising Therapeutic Targets for Treatment of Rheumatoid Arthritis. *Front. Immunol.* **12**, 686155 (2021). 34305919 10.3389/fimmu.2021.686155 PMC8299711

[5] Yin, Y. et al. Structure-based design and synthesis of 1H-pyrazolo[3,4-d]pyrimidin-4-amino derivatives as Janus kinase 3 inhibitors. *Bioorg. Med. Chem.* **26** (17), 4774–4786 (2018). 30139575 10.1016/j.bmc.2018.04.005

[6] Shu, L. et al. Design, synthesis, and pharmacological evaluation of 4- or 6-phenyl-pyrimidine derivatives as novel and selective Janus kinase 3 inhibitors. *Eur. J. Med. Chem.* **191**, 112148 (2020). 32097841 10.1016/j.ejmech.2020.112148

[7] Lin, C. M., Cooles, F. A. & Isaacs, J. D. Basic Mechanisms of JAK Inhibition. *Mediterr. J. Rheumatol.* **31** (Suppl 1), 100–104 (2020). 32676567 10.31138/mjr.31.1.100 PMC7361186

[8] Wang, X. et al. Combination of Synonymous and Missense Mutations in JAK3 Gene Contributes to Severe Combined Immunodeficiency in One Child. *Hum. Mutat.* **2023**, 6633251 (2023). 40225147 10.1155/2023/6633251 PMC11919225

[9] Chen, C. et al. A highly selective JAK3 inhibitor is developed for treating rheumatoid arthritis by suppressing γc cytokine–related JAK-STAT signal. *Sci. Adv.* **8** (33), eabo4363 (2022). 35984890 10.1126/sciadv.abo4363 PMC9390995

[10] Hamaguchi, H. et al. Discovery and structural characterization of peficitinib (ASP015K) as a novel and potent JAK inhibitor. *Bioorg. Med. Chem.* **26** (18), 4971–4983 (2018). 30145050 10.1016/j.bmc.2018.08.005

[11] King, B. et al. Efficacy and safety of ritlecitinib in adults and adolescents with alopecia areata: a randomised, double-blind, multicentre, phase 2b-3 trial. *Lancet* **401** (10387), 1518–1529 (2023). 37062298 10.1016/S0140-6736(23)00222-2

[12] Genovese, M. C., Yang, F., Østergaard, M. & Kinnman, N. Efficacy of VX-509 (decernotinib) in combination with a disease-modifying antirheumatic drug in patients with rheumatoid arthritis: clinical and MRI findings. *Ann. Rheum. Dis.* **75** (11), 1979–1983 (2016). 27084959 10.1136/annrheumdis-2015-208901

[13] Pei, H. et al. Discovery of a highly selective JAK3 inhibitor for the treatment of rheumatoid arthritis. *Sci. Rep.* **8** (1), 5273 (2018). 29588471 10.1038/s41598-018-23569-y PMC5869712

[14] Fang, Z. et al. CS12192, a novel JAK3/JAK1/TBK1 inhibitor, synergistically enhances the anti-inflammation effect of methotrexate in a rat model of rheumatoid arthritis. *Int. J. Mol. Sci.* **23**(21). (2022). 10.3390/ijms232113394 PMC9658750 36362183

[15] Tan, L. et al. Development of Selective Covalent Janus Kinase 3 Inhibitors. *J. Med. Chem.* **58** (16), 6589–6606 (2015). 26258521 10.1021/acs.jmedchem.5b00710 PMC4777322

[16] Lin, X., Li, X. & Lin, X. A review on applications of computational methods in drug screening and design. *Molecules* **25**(6). (2020). 10.3390/molecules25061375 PMC7144386 32197324

[17] Wang, X., Song, K., Li, L. & Chen, L. Structure-Based Drug Design Strategies and Challenges. *Curr. Top. Med. Chem.* **18** (12), 998–1006 (2018). 30101712 10.2174/1568026618666180813152921

[18] Santos, L. H. S., Ferreira, R. S. & Caffarena, E. R. Integrating Molecular Docking and Molecular Dynamics Simulations. *Methods Mol. Biol.* **2053**, 13–34 (2019). 31452096 10.1007/978-1-4939-9752-7_2

[19] Lavecchia, A. & Di Giovanni, C. Virtual screening strategies in drug discovery: a critical review. *Curr. Med. Chem.* **20** (23), 2839–2860 (2013). 23651302 10.2174/09298673113209990001

[20] Faris, A., Hadni, H., Ibrahim, I. M. & Elhallaoui, M. In silico discovery of potent and selective Janus kinase 3 (JAK3) inhibitors through 3D-QSAR, covalent docking, ADMET analysis, molecular dynamics simulations, and binding free energy of pyrazolopyrimidine derivatives. *J. Biomol. Struct. Dynamics*. **42** (9), 4817–4833 (2024). 10.1080/07391102.2023.2222839 37338041

[21] Li, Y. et al. Design of rational JAK3 inhibitors based on the parent core structure of 1, 7-dihydro-dipyrrolo [2, 3-b: 3′, 2′-e] pyridine. *Int. J. Mol. Sci.* **23** (10), 5437 (2022). 35628248 10.3390/ijms23105437 PMC9141313

[22] Prieto, M., Niño, A., Acosta-Guzmán, P. & Guevara-Pulido, J. Design and synthesis of a potential selective JAK-3 inhibitor for the treatment of rheumatoid arthritis using predictive QSAR models. *Inf. Med. unlocked*. **45**, 101464 (2024).

[23] Banerjee, K., Chandrasekar, B., Sathish, S., Sohn, H. & Madhavan, T. Computational drug repositioning for IL6 triggered JAK3 in rheumatoid arthritis using FDA database. *Mol. Diversity*. **29** (3), 2049–2061 (2025). 10.1007/s11030-024-10958-x 39141207

[24] Gehringer, M., Forster, M. & Laufer, S. A. Solution-phase parallel synthesis of ruxolitinib-derived Janus kinase inhibitors via copper-catalyzed azide-alkyne cycloaddition. *ACS Comb. Sci.* **17** (1), 5–10 (2015). 25405713 10.1021/co500122h

[25] Flanagan, M. E. et al. Discovery of CP-690,550: a potent and selective Janus kinase (JAK) inhibitor for the treatment of autoimmune diseases and organ transplant rejection. *J. Med. Chem.* **53** (24), 8468–8484 (2010). 21105711 10.1021/jm1004286

[26] Abbasi, M., Sadeghi-Aliabadi, H. & Amanlou, M. 3D-QSAR, molecular docking, and molecular dynamic simulations for prediction of new Hsp90 inhibitors based on isoxazole scaffold. *J. Biomol. Struct. Dyn.* **36** (6), 1463–1478 (2018). 28482755 10.1080/07391102.2017.1326319

[27] Bahia, M. S. et al. A comparison between 2D and 3D descriptors in QSAR modeling based on bio-active conformations. *Mol. Inf.* **42** (4), 2200186 (2023). 10.1002/minf.202200186 36617991

[28] Xie, D. et al. 3D-QSAR, design, molecular docking and dynamics simulation studies of novel 6-hydroxybenzothiazole-2-carboxamides as potentially potent and selective monoamine oxidase B inhibitors. *Front. Pharmacol.* **16**, 1545791 (2025). 39981188 10.3389/fphar.2025.1545791 PMC11841475

[29] SYBYL-X 2.0 (Tripos Inc. St. Louis U. SYBYL-X 2.0 (Tripos Inc. St. Louis, USA).

[30] Dörgő, G., Péter Hamadi, O., Varga, T. & Abonyi, J. Mixtures of QSAR models: Learning application domains of p K predicto rs. *J. Chemom.* **34** (4), e3223 (2020).

[31] Zhang, Y-K. et al. Design and evaluation of piperidine carboxamide derivatives as potent ALK inhibitors through 3D-QSAR modeling, artificial neural network and computational analysis. *Arab. J. Chem.* **17** (9), 105863 (2024).

[32] Maestro, S. & New York, L. L. C. NY, Maestro,Schrödinger, LLC, New York, NY (2015).

[33] Abraham, M. J. et al. GROMACS: High performance molecular simulations through multi-level parallelism from laptops to supercomputers. *SoftwareX* **1–2**, 19–25 (2015).

[34] Pettersen, E. F. et al. UCSF Chimera–a visualization system for exploratory research and analysis. *J. Comput. Chem.* **25** (13), 1605–1612 (2004). 15264254 10.1002/jcc.20084

[35] Sousa da Silva, A. W. & Vranken, W. F. ACPYPE - AnteChamber PYthon Parser interfacE. *BMC Res. Notes*. **5** (1), 367 (2012). 22824207 10.1186/1756-0500-5-367 PMC3461484

[36] Papaleo, E., Mereghetti, P., Fantucci, P., Grandori, R. & De Gioia, L. Free-energy landscape, principal component analysis, and structural clustering to identify representative conformations from molecular dynamics simulations: the myoglobin case. *J. Mol. Graph. Model.* **27** (8), 889–899 (2009). 19264523 10.1016/j.jmgm.2009.01.006

[37] Lanka, G., Banerjee, S., Regula, S., Adhikari, N. & Ghosh, B. Pharmacophore modeling, 3D-QSAR, and MD simulation-based overture for the discovery of new potential HDAC1 inhibitors. *J. Biomol. Struct. Dyn. *1–24. (2024). 10.1080/07391102.2024.2429020 39587443

[38] Ouyang, L. et al. Combined structure-based pharmacophore and 3D-QSAR studies on phenylalanine series compounds as TPH1 inhibitors. *Int. J. Mol. Sci.* **13** (5), 5348–5363 (2012). 22754301 10.3390/ijms13055348 PMC3382768

[39] Chauhan, J. S. et al. QSAR-based models for designing quinazoline/imidazothiazoles/pyrazolopyrimidines based inhibitors against wild and mutant EGFR. *PloS one*. **9** (7), e101079 (2014). 24992720 10.1371/journal.pone.0101079 PMC4081576

[40] Lorca, M. et al. 2D/3D-QSAR model development based on a quinoline pharmacophoric core for the inhibition of Plasmodium falciparum: an in silico approach with experimental validation. *Pharmaceuticals* **17** (7), 889 (2024). 39065740 10.3390/ph17070889 PMC11279914

[41] Yazdani, B. et al. Discovery of novel direct small-molecule inhibitors targeting HIF-2α using structure-based virtual screening, molecular dynamics simulation, and MM-GBSA calculations. *Mol. Diversity*. **28** (3), 1203–1224 (2024). 10.1007/s11030-023-10650-6 37120484

[42] Jolliffe, I. T. & Cadima, J. Principal component analysis: a review and recent developments. *Philos. Trans. R. Soc. A Math. Phys. Eng. Sci.* **374**(2065), 20150202 (2016). 10.1098/rsta.2015.0202 PMC4792409 26953178

[43] Shaw, D. E. et al. (eds) Millisecond-scale molecular dynamics simulations on Anton. In *Proceedings of the conference on high performance computing networking, storage and analysis*; (2009).

