# Supplementary material for: 3D-QSAR and free energy landscape analysis for predicting novel JAK3 inhibitors in rheumatoid arthritis therapy
Source: Sci Rep. 2026 Apr 29;16:19835. doi: 10.1038/s41598-026-50981-6 (PMC13315631; doi:10.1038/s41598-026-50981-6)
Supplement: Supplementary file 1 — Supplementary Material 1 [file 41598_2026_50981_MOESM1_ESM.docx]

**Table S1**. The molecular structures of studied compounds.

**A B C D**

| Comp. | R_1_ | R_2_ | R_3_ | R_4_ | X |
| --- | --- | --- | --- | --- | --- |
|  |  |  |  |  |  |
| **1 (A)** |  | H | H |  | - |
| **2 (A)** |  | H | H |  | - |
| **3 (A)** |  | H | H |  | - |
| **4 (A)** |  | H | H |  | - |
| **5 (A)** |  | H | H |  | - |
| **6 (A)** |  | H | H |  | - |
| **7 (A)** |  | H |  | H | - |
| **8 (A)** |  | H |  | H | - |
| **9 (A)** | H |  | H |  | - |
| **10 (A)** | H |  | H |  | - |
| **11 (A)** | H |  |  | H | - |
| **12 (A)** | H |  |  | H | - |
| **13 (A)** |  | H | H |  | - |
| **14 (A)** |  | H | H |  | - |
| **15 (A)** |  | H | H |  | - |
| **16 (A)** |  | H | H |  | - |
| **17 (A)** |  | H | H |  | - |
| **18 (A)** |  | H | H |  | - |
| **19 (A)** |  | H | H |  | - |
| **20 (A)** |  | H | H |  | - |
| **21 (A)** |  | H | H |  | - |
| **22 (A)** |  | H | H |  | - |
| **23 (A)** |  | H | H |  | - |
| **24 (A)** |  | H | H |  | - |
| **25 (A)** |  | H | H |  | - |
| **26 (A)** |  | H | H |  | - |
| **27 (A)** |  | H | H |  | - |
| **28 (A)** |  | H | H |  | - |
| **29 (A)** |  | H | H |  | - |
| **30 (A)** |  | H |  | H | - |
| **31 (A)** |  | H |  | H | - |
| **32 (A)** | H |  | H |  | - |
| **33 (A)** | H |  | H |  | - |
| **34 (A)** | H |  | H |  | - |
| **35 (A)** | H |  | H |  | - |
| **36 (A)** | H |  |  | H | - |
| **37 (A)** | H |  |  | H | - |
| **38 (A)** | H |  |  | H | - |
| **39 (A)** | H |  |  | H | - |
| **2a (B)** |  | - | - | - | - |
| **2b (B)** |  | - | - | - | - |
| **2c (B)** |  | - | - | - | - |
| **2d (B)** |  | - | - | - | - |
| **2e (B)** |  | - | - | - | - |
| **2f (B)** |  | - | - | - | - |
| **2g (B)** |  | - | - | - | - |
| **2h (B)** |  | - | - | - | - |
| **2i (B)** |  | - | - | - | - |
| **6a (C)** | H |  | - | - | - |
| **6b (C)** |  | H | - | - | - |
| **6c (C)** |  |  | - | - | - |
| **6d (C)** |  | H | - | - | - |
| **6e (C)** |  | H | - | - | - |
| **6f (C)** |  |  | - | - | - |
| **6g (C)** |  | H | - | - | - |
| **6h (C)** |  | H | - | - | - |
| **16a (C)** |  |  | - | - | - |
| **16b (C)** |  |  | - | - | - |
| **16c (C)** |  |  | - | - | - |
| **16d (C)** |  |  | - | - | - |
| **16e (C)** |  |  | - | - | - |
| **17a (C)** |  |  | - | - | N |
| **17b (C)** |  |  | - | - | N |
| **17c (C)** |  |  | - | - | N |
| **17d (C)** |  |  | - | - | N |
| **18a (C)** |  |  | - | - | N |
| **18b (C)** |  |  | - | - | N |
| **18c (C)** |  |  | - | - | N |
| **18d (C)** |  |  | - | - | N |
| **18e (C)** |  |  | - | - | N |
| **18f (C)** |  |  | - | - | N |
| **18g (C)** |  |  | - | - | N |
| **18h (C)** |  |  | - | - | N |
| **18i (C)** |  |  | - | - | N |
| **18j (C)** |  |  | - | - | N |
| **18k (C)** |  |  | - | - | N |
| **18L (C)** |  |  | - | - | N |
| **18m (C)** |  |  | - | - | N |
| **2-1 (D)** |  | - | - | - | - |
| **2-3 (D)** |  | - | - | - | - |
| **2-11 (D)** |  | - | - | - | - |
| **2-12 (D)** |  | - | - | - | - |
| **2-18 (D)** |  | - | - | - | - |
| **2-19 (D)** |  | - | - | - | - |
| **2-20 (D)** |  | - | - | - | - |
| **2-21 (D)** |  | - | - | - | - |
| **2-22 (D)** |  | - | - | - | - |
| **2-24 (D)** |  | - | - | - | - |
| **2-25 (D)** |  | - | - | - | - |
| **2-27 (D)** |  | - | - | - | - |
| **2-28 (D)** |  | - | - | - | - |
| **2-31 (D)** |  | - | - | - | - |
| **2-34 (D)** |  | - | - | - | - |
| **2-36 (D)** |  | - | - | - | - |
| 2-38 (D) |  | - | - | - | - |

**Table S2.** Observed (experimental) and predicted pIC_50_ values derived from CoMFA and CoMSIA analyses.

| Comp. | Actual pIC_50_ | CoMFA  predicted | Residual | CoMSIA predicted | Residual | Comp. | Actual pIC_50_ | CoMFA  predicted | Residual | CoMSIA predicted | Residual |
| --- | --- | --- | --- | --- | --- | --- | --- | --- | --- | --- | --- |
| **1 (A)** | 6.519 | 6.571 | -0.052 | 6.679 | -0.16 | **6a (C)** | 6.86 | 7.176 | -0.316 | 7.039 | -0.179 |
| **2 (A)** | 6.996 | 6.779 | 0.217 | 6.731 | 0.265 | **6b (C)** | 7.187 | 7.125 | 0.062 | 6.925 | 0.262 |
| **3 (A)** | 6.738 | 6.771 | -0.033 | 6.829 | -0.091 | ***6c (C)** | 7.699 | 7.373 | 0.326 | 7.129 | 0.57 |
| ***4 (A)** | 6.51 | 6.58 | -0.07 | 6.484 | 0.026 | **6d (C)** | 7.215 | 7.031 | 0.184 | 6.994 | 0.221 |
| **5 (A)** | 6.86 | 7.109 | -0.249 | 6.971 | -0.111 | **6e (C)** | 6.097 | 6.783 | -0.686 | 7.031 | -0.934 |
| **6 (A)** | 6.223 | 6.56 | -0.337 | 6.694 | -0.471 | **6f (C)** | 5.699 | 5.86 | -0.161 | 6.027 | -0.328 |
| **7 (A)** | 7.26 | 7.186 | 0.074 | 7.543 | -0.283 | ***6g (C)** | 6.638 | 7.329 | -0.691 | 7.2 | -0.562 |
| **8 (A)** | 7.523 | 7.661 | -0.138 | 7.321 | 0.202 | **6h (C)** | 6.268 | 6.505 | -0.237 | 6.598 | -0.33 |
| ***9 (A)** | 7.174 | 7.138 | 0.036 | 7.131 | 0.043 | **16a (C)** | 7.699 | 7.636 | 0.063 | 7.517 | 0.182 |
| **10 (A)** | 6.951 | 7.093 | -0.142 | 7.332 | -0.381 | **16b (C)** | 7.495 | 7.603 | -0.108 | 7.864 | -0.369 |
| ***11 (A)** | 8.041 | 8.157 | -0.116 | 7.806 | 0.235 | **16c (C)** | 7.42 | 7.624 | -0.204 | 7.858 | -0.438 |
| **12 (A)** | 8.77 | 8.88 | -0.11 | 8.531 | 0.239 | **16d (C)** | 7.886 | 8.04 | -0.154 | 7.924 | -0.038 |
| ***13 (A)** | 6.246 | 6.439 | -0.193 | 6.362 | -0.116 | **16e (C)** | 6.824 | 6.939 | -0.115 | 7.269 | -0.445 |
| **14 (A)** | 6.839 | 6.8 | 0.039 | 6.964 | -0.125 | **17a (C)** | 7.854 | 8.222 | -0.368 | 7.459 | 0.395 |
| **15 (A)** | 7.538 | 7.55 | -0.012 | 7.088 | 0.45 | **17b (C)** | 7.585 | 7.78 | -0.195 | 7.48 | 0.105 |
| **16 (A)** | 7.187 | 7.098 | 0.089 | 6.996 | 0.191 | **17c (C)** | 7.377 | 7.519 | 0.858 | 7.22 | 1.157 |
| **17 (A)** | 7.456 | 7.626 | -0.17 | 7.274 | 0.182 | ***17d (C)** | 7.495 | 7.587 | -0.092 | 7.498 | -0.003 |
| ***18 (A)** | 6.77 | 6.759 | 0.011 | 6.703 | 0.067 | **18a (C)** | 6.319 | 6.467 | -0.148 | 6.944 | -0.625 |
| **19 (A)** | 7.174 | 7.523 | -0.349 | 6.984 | 0.19 | **18b (C)** | 6.932 | 7.098 | -0.166 | 6.959 | -0.027 |
| **20 (A)** | 7.036 | 7.286 | -0.25 | 6.929 | 0.107 | **18c (C)** | 6.638 | 7.029 | -0.391 | 7.118 | -0.48 |
| ***21 (A)** | 6.656 | 6.783 | -0.127 | 6.691 | -0.035 | **18d (C)** | 7.538 | 7.709 | -0.171 | 7.229 | 0.309 |
| **22 (A)** | 6.614 | 7.018 | -0.404 | 6.967 | -0.353 | **18e (C)** | 6.845 | 7.117 | -0.272 | 7.14 | -0.295 |
| **23 (A)** | 7.022 | 7.046 | -0.024 | 6.955 | 0.067 | ***18f (C)** | 7.398 | 7.482 | -0.084 | 7.445 | -0.047 |
| ***24 (A)** | 7.18 | 6.953 | 0.227 | 6.963 | 0.217 | **18g (C)** | 7.409 | 7.339 | 0.07 | 7.183 | 0.226 |
| **25 (A)** | 6.967 | 7.159 | -0.192 | 6.991 | -0.024 | **18h (C)** | 6.833 | 7.088 | -0.255 | 7.244 | -0.411 |
| **26 (A)** | 6.987 | 7.281 | -0.294 | 6.976 | 0.011 | **18i (C)** | 7.699 | 7.214 | 0.485 | 7.132 | 0.567 |
| **27 (A)** | 6.426 | 6.357 | 0.069 | 6.624 | -0.198 | **18j (C)** | 7.721 | 7.782 | -0.061 | 7.273 | 0.448 |
| **28 (A)** | 6.597 | 6.487 | 0.11 | 6.637 | -0.04 | **18k (C)** | 6.947 | 7.09 | -0.143 | 7.161 | -0.214 |
| **29 (A)** | 6.896 | 7.004 | -0.108 | 6.885 | 0.011 | **18l (C)** | 7.886 | 8.038 | -0.152 | 7.302 | 0.584 |
| **30 (A)** | 7.481 | 7.76 | -0.279 | 7.332 | 0.149 | **18m(C)** | 8.481 | 8.586 | -0.105 | 7.318 | 1.163 |
| **31 (A)** | 6.818 | 7.122 | -0.304 | 7.239 | -0.421 | **2-1 (D)** | 5.004 | 5.054 | -0.05 | 5.09 | -0.086 |
| **32 (A)** | 7.119 | 7.264 | -0.145 | 7.188 | -0.069 | **2-3 (D)** | 5.112 | 5.074 | 0.038 | 5.04 | 0.072 |
| **33 (A)** | 7.066 | 7.163 | -0.097 | 7.203 | -0.137 | ***2-11 (D)** | 5.002 | 5.178 | -0.176 | 5.133 | -0.131 |
| ***34 (A)** | 7.004 | 6.867 | 0.137 | 6.987 | 0.017 | **2-12 (D)** | 5.24 | 5.025 | 0.215 | 5.155 | 0.085 |
| **35 (A)** | 7.108 | 7.264 | -0.156 | 7.136 | -0.028 | **2-18 (D)** | 5.431 | 5.408 | 0.023 | 5.313 | 0.118 |
| **36 (A)** | 7.854 | 8.05 | -0.196 | 7.794 | 0.06 | **2-19 (D)** | 6.137 | 6.18 | -0.043 | 5.573 | 0.564 |
| **37 (A)** | 7.62 | 7.885 | -0.265 | 7.811 | -0.191 | **2-20 (D)** | 5.028 | 4.918 | 0.11 | 5.334 | -0.306 |
| **38 (A)** | 8.215 | 8.443 | -0.228 | 7.836 | 0.379 | **2-21 (D)** | 5 | 5.152 | -0.152 | 5.365 | -0.365 |
| **39 (A)** | 7.824 | 8.087 | -0.263 | 7.819 | 0.005 | **2-22 (D)** | 5.575 | 5.476 | 0.099 | 5.293 | 0.282 |
| **2a (B)** | 6.678 | 6.831 | -0.153 | 6.457 | 0.221 | **2-24 (D)** | 5.492 | 5.398 | 0.094 | 5.206 | 0.286 |
| **2b (B)** | 6.678 | 6.568 | 0.11 | 6.324 | 0.354 | **2-25 (D)** | 5.303 | 5.343 | -0.04 | 5.159 | 0.144 |
| **2c (B)** | 6.569 | 6.595 | -0.026 | 6.459 | 0.11 | **2-27 (D)** | 5.394 | 5.497 | -0.103 | 5.056 | 0.338 |
| **2d (B)** | 6.456 | 6.569 | -0.113 | 6.439 | 0.017 | ***2-28 (D)** | 5.388 | 5.224 | 0.164 | 5.208 | 0.18 |
| ***2e (B)** | 6.409 | 7.066 | -0.657 | 6.796 | -0.387 | **2-31 (D)** | 5.058 | 4.946 | 0.112 | 5.263 | -0.205 |
| **2f (B)** | 6.796 | 6.967 | -0.171 | 6.696 | 0.1 | **2-34 (D)** | 5.002 | 5.21 | -0.208 | 5.588 | -0.586 |
| **2g (B)** | 6.432 | 7.019 | -0.587 | 6.761 | -0.329 | **2-36 (D)** | 5.648 | 5.573 | 0.075 | 5.357 | 0.291 |
| **2h (B)** | 6.959 | 7.264 | -0.305 | 7.069 | -0.11 | **2-38 (D)** | 5.021 | 5.079 | -0.058 | 5.176 | -0.155 |
| ***2i (B)** | 7.237 | 7.378 | -0.141 | 7.078 | 0.159 |  |  |  |  |  |  |

**Table S3.** Statistical Parameters of the 31 COMSIA Models

| Model | R2 | Q2 | ONC | SEE |
| --- | --- | --- | --- | --- |
| S | 0.688 | 0.547 | 2 | 0.508 |
| E | 0.64 | 0.532 | 1 | 0.542 |
| H | 0.854 | 0.637 | 3 | 0.349 |
| D | 0.335 | 0.193 | 4 | 0.751 |
| A | 0.893 | 0.601 | 6 | 0.305 |
| SE | 0.932 | 0.626 | 6 | 0.243 |
| SH | 0.928 | 0.72 | 6 | 0.25 |
| SD | 0.8 | 0.686 | 3 | 0.409 |
| SA | 0.868 | 0.615 | 4 | 0.335 |
| EH | 0.929 | 0.645 | 5 | 0.246 |
| ED | 0.91 | 0.661 | 6 | 0.28 |
| EA | 0.783 | 0.602 | 2 | 0.423 |
| HD | 0.822 | 0.695 | 3 | 0.385 |
| HA | 0.898 | 0.688 | 4 | 0.294 |
| DA | 0.804 | 0.651 | 4 | 0.407 |
| SHE | 0.903 | 0.666 | 4 | 0.287 |
| SED | 0.854 | 0.687 | 3 | 0.35 |
| SEA | 0.804 | 0.629 | 2 | 0.402 |
| SHD | 0.845 | 0.721 | 3 | 0.36 |
| SHA | 0.894 | 0.7 | 4 | 0.3 |
| SDA | 0.836 | 0.704 | 3 | 0.37 |
| EHD | 0.893 | 0.691 | 4 | 0.3 |
| EHA | 0.947 | 0.67 | 6 | 0.214 |
| EDA | 0.864 | 0.668 | 4 | 0.339 |
| HAD | 0.85 | 0.719 | 3 | 0.354 |
| SEHD | 0.869 | 0.704 | 3 | 0.331 |
| SEHA | 0.905 | 0.674 | 4 | 0.283 |
| SEDA | 0.853 | 0.69 | 3 | 0.351 |
| EHDA | 0.863 | 0.704 | 3 | 0.339 |
| SHDA | 0.856 | 0.725 | 3 | 0.347 |
| SEHDA | 0.865 | 0.705 | 3 | 0.336 |

**Table S4**. Docking results of the template and designed compounds on JAK3.

| **Compound** | **XP-GScore** | **Hydrogen bond interactions (Distance Å)** | **The main Amino acids in the active site** |
| --- | --- | --- | --- |
| **12 (A)** | -9.804 | Leu905(2.2,3.3), Lys855(2.2) | Leu905, Lys855 |
| **79** | -14.162 | Cys909(1.81), Asp912(1.76), Leu905(1.84,2.09), Lys855(2.09) | Cys909, Asp912, Leu905, Lys855 |
| **9** | -13.219 | Cys909(2.00), Asp912(1.61), Leu905(2.06,2.26), Tyr904(2.05), Lys855(2.20) | Cys909, Asp912, Leu905, Tyr904, Lys855 |
| **54** | -13.050 | Cys909(1.94), Asp912(1.60), Leu905(1.98,2.34), Asp967(2.71) | Cys909, Asp912, Leu905, Asp967 |
| **10** | -12.893 | Cys909(2.03), Tyr904(2.04), Leu905(1.93,2.25), Asp912(1.60), Lys855(2.20), | Cys909, Tyr904, Leu905, Asp912, Lys855 |
| **84** | -12.762 | Glu903(2.13), Leu905(1.85,2.02), Cys909(1.93), Asp912(1.79) | Glu903, Leu905, Cys909, Asp912 |
| **90** | -12.694 | Glu903(2.37), Leu905(1.71,2.02), Cys909(1.85), Asp912(1.89), | Glu903, Leu905, Cys909, Asp912, Glu871* |
| **55** | -12.658 | Leu905(2.01,2.36), Cys909(1.87), Asp912(1.65), Asp967(2.76) | Leu905, Cys909, Asp912, Asp967 |
| **86** | -12.599 | Leu905(1.93,2.12), Cys909(1.87), Asp912(1.63), Lys855(2.19), | Leu905, Cys909, Asp912, Lys855, Glu871* |

The ionic interaction is shown with *.


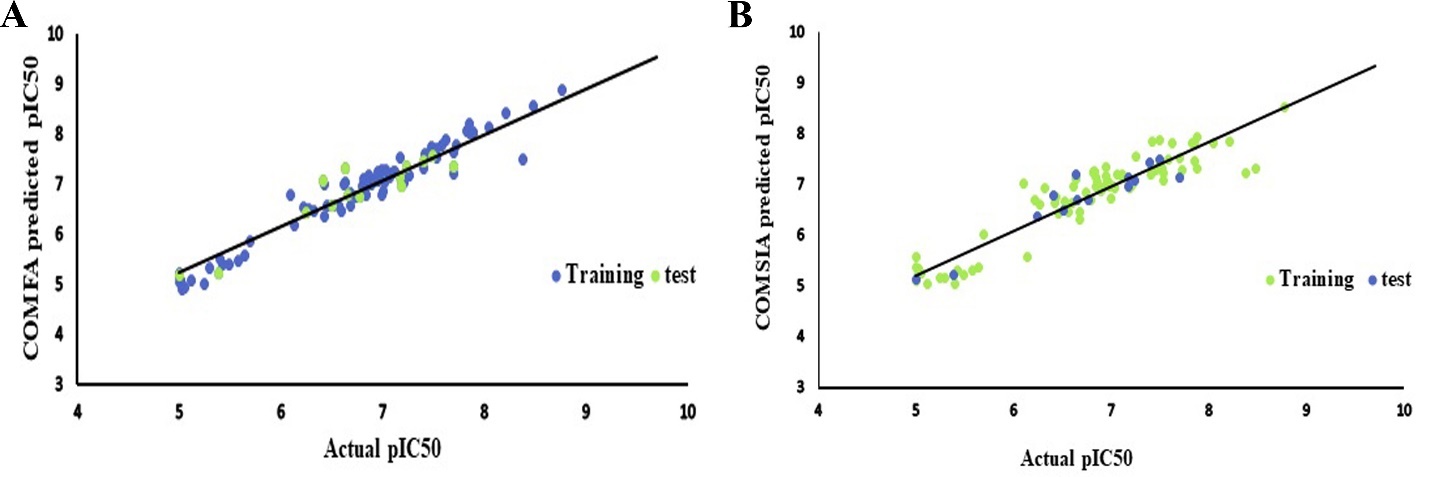


**Figure S1.** Graphs comparing the predicted pIC_50_ values against the actual values for all molecules: A) CoMFA, model, training (blue) and test, B) CoMSIA.model, training and test (blue).


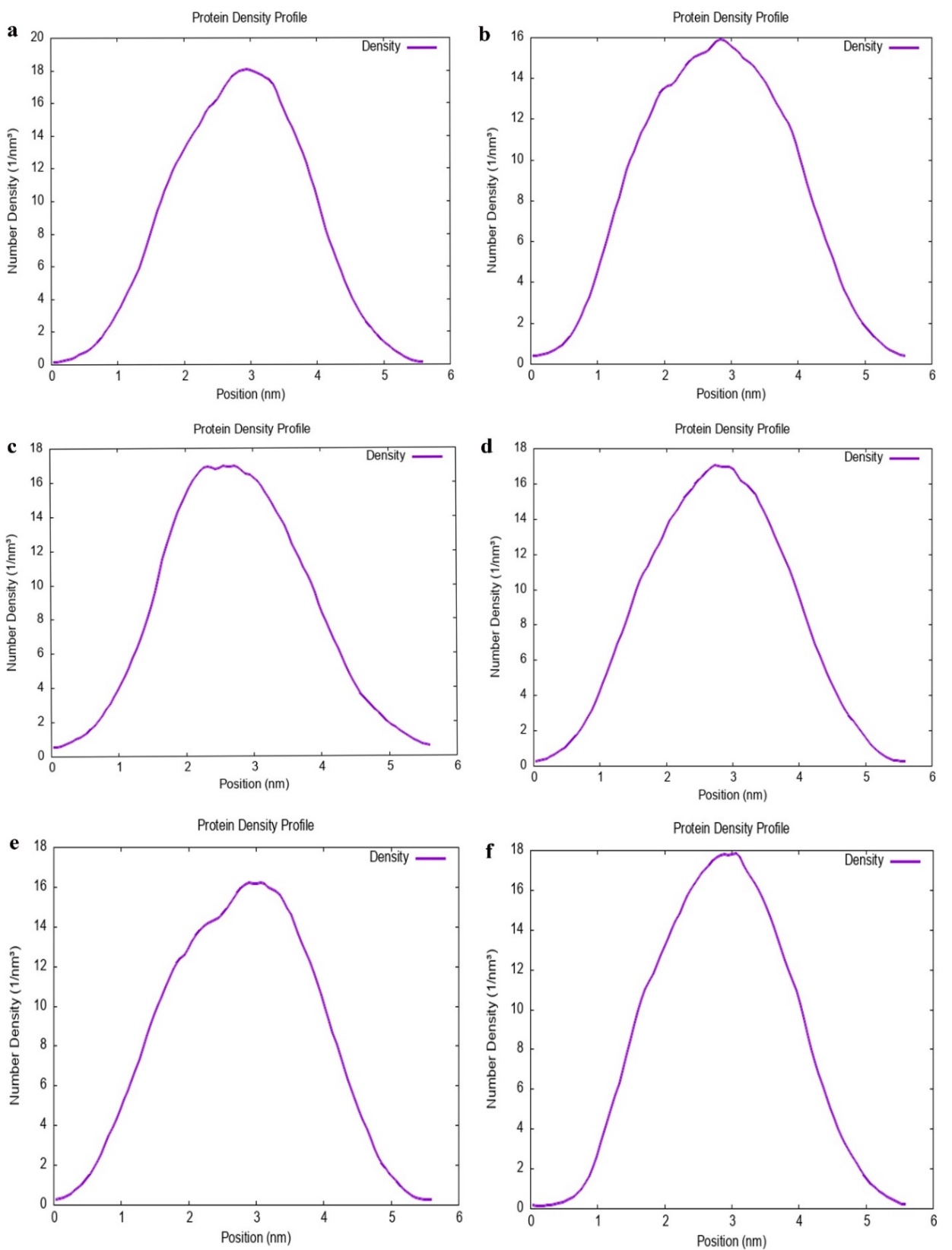


**Figure S2.** The Probability density function (PDF) of JAK3 in complex with ligands for 100 ns in the MD simulation study. a) JAK3-12(A) complex, b) JAK3-2 complex, c) JAK3-55 complex, d) JAK3-56 complex, e) JAK3-79 complex, and f) JAK3-85 complex
